# Rational Development of Guanidinate and Amidinate Based Cerium and Ytterbium Complexes as Atomic Layer Deposition Precursors: Synthesis, Modeling, and Application

**DOI:** 10.1002/chem.202003907

**Published:** 2021-01-20

**Authors:** Parmish Kaur, Lukas Mai, Arbresha Muriqi, David Zanders, Ramin Ghiyasi, Muhammad Safdar, Nils Boysen, Manuela Winter, Michael Nolan, Maarit Karppinen, Anjana Devi

**Affiliations:** ^1^ Inorganic Materials Chemistry Ruhr University Bochum Universitätsstraße 150 44801 Bochum Germany; ^2^ Tyndall National Institute University College Cork Lee Maltings Cork T12 R5CP Ireland; ^3^ Department of Chemistry and Materials Science Aalto University Kemistintie 1 00076 Aalto Espoo Finland

**Keywords:** atomic layer deposition, cerium oxide, DFT calculations, precursors, thermal analysis

## Abstract

Owing to the limited availability of suitable precursors for vapor phase deposition of rare‐earth containing thin‐film materials, new or improved precursors are sought after. In this study, we explored new precursors for atomic layer deposition (ALD) of cerium (Ce) and ytterbium (Yb) containing thin films. A series of homoleptic tris‐guanidinate and tris‐amidinate complexes of cerium (Ce) and ytterbium (Yb) were synthesized and thoroughly characterized. The C‐substituents on the N‐C‐N backbone (Me, NMe_2_, NEt_2_, where Me=methyl, Et=ethyl) and the N‐substituents from symmetrical iso‐propyl (*i*Pr) to asymmetrical tertiary‐butyl (*t*Bu) and Et were systematically varied to study the influence of the substituents on the physicochemical properties of the resulting compounds. Single crystal structures of [Ce(dpdmg)_3_] **1** and [Yb(dpdmg)_3_] **6** (dpdmg=*N,N'*‐diisopropyl‐2‐dimethylamido‐guanidinate) highlight a monomeric nature in the solid‐state with a distorted trigonal prismatic geometry. The thermogravimetric analysis shows that the complexes are volatile and emphasize that increasing asymmetry in the complexes lowers their melting points while reducing their thermal stability. Density functional theory (DFT) was used to study the reactivity of amidinates and guanidinates of Ce and Yb complexes towards oxygen (O_2_) and water (H_2_O). Signified by the DFT calculations, the guanidinates show an increased reactivity toward water compared to the amidinate complexes. Furthermore, the Ce complexes are more reactive compared to the Yb complexes, indicating even a reactivity towards oxygen potentially exploitable for ALD purposes. As a representative precursor, the highly reactive [Ce(dpdmg)_3_] **1** was used for proof‐of‐principle ALD depositions of CeO_2_ thin films using water as co‐reactant. The self‐limited ALD growth process could be confirmed at 160 °C with polycrystalline cubic CeO_2_ films formed on Si(100) substrates. This study confirms that moving towards nitrogen‐coordinated rare‐earth complexes bearing the guanidinate and amidinate ligands can indeed be very appealing in terms of new precursors for ALD of rare earth based materials.

## Graphical abstract

**Systematic engineering** of Ce and Yb based amidinate and guanidinate complexes resulted in precursors suitable for atomic layer deposition (ALD) applications. Computational modeling reveals the reactivity of the complexes towards water and oxygen. A water assisted ALD process for the growth of CeO_2_ thin films was demonstrated with the Ce tris‐guanidinate precursor.

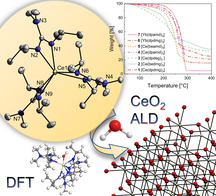

## Introduction

Rare earth (RE) metal containing materials are very interesting in different fields of applications ranging from optical coatings,^[1]^ optical waveguides,^[2]^ catalysis,^[3]^ protective coatings,^[4]^ fuel cells^[5]^ to high‐κ materials^[6]^ in the microelectronic industry. In particular, cerium oxide (CeO_2_) is encouraging for catalysis,^[7]^ water splitting,^[8]^ solid oxide fuel cells,^[9]^ protective coatings,^[10]^ and is also considered as a possible high‐κ gate dielectric material in complementary metal‐oxide‐semiconductor devices.^[11]^ Yb containing films are finding increasing attention in the advancement of semiconductor devices^[12]^ with ytterbium‐doped optical fibers being relevant for high power laser applications.^[13]^ Thus, recently the interest in the growth of high‐quality RE‐based materials^[14]^ has been on the rise, particularly for conformal coatings with a precise tunable thickness on complex architectures which can be obtained by atomic layer deposition (ALD).^[15]^

ALD is a powerful technique that uses a self‐limiting growth mechanism by employing pulses of a gaseous chemical metal‐organic compound (precursor) and a suitable co‐reactant for the desired thin film material which are separated by inert gas purges to ensure layer‐by‐layer growth of conformal, uniform and pinhole‐free films.^[16]^ Due to the unique surface saturation caused by the chemical surface reactions, the ALD process is strongly dependent on the chemical properties of the employed metal precursor.^[16a]^ Therefore, ALD precursors must fulfill several requirements. First, they should be reactive towards the substrate surface and the co‐reactant. Secondly, they should be volatile to be brought into the gas phase and thermally stable for a prolonged time at the chosen evaporation temperature.^[17]^ Thirdly, they should at least be thermally stable on the time scale of an ALD cycle to prevent their decomposition and uncontrollable reactions at given deposition temperatures. Naturally, long‐term stability at these temperatures is a practical feature. Additionally, a precursor in the liquid state is advantageous as it can provide a reproducible rate of vaporization more likely than a solid one. From a chemistry point of view, these properties can be tuned by modifying the ligand of a metal‐organic complex, to meet the demands of the process. Typically, ligands^[18]^ such as β‐diketonates,^[19]^ cyclopentadienyls,^[20]^ alkoxides,^[21]^ bis(trimethylsilyl)amides,^[22]^ amidinates^[23]^ and guanidinates^[24]^ are used in the case of rare‐earth metals. Since the majority of available RE precursors do not satisfy one or more requirements described above, the deposition process can be affected negatively so that the properties of the deposited films do not match the desired specifications. The number of reports on the suitable precursors for rare earth metals particularly for cerium and ytterbium, is limited in comparison to other metals.

Early‐generation precursors such as RE‐alkoxides have been demonstrated to exhibit poor volatility as they tend to oligomerize and therefore, could only be applied successfully in liquid injection delivery ALD systems^[25]^ as shown for [Ce(mmp)_4_] (mmp=1‐methoxy‐ 2‐methyl‐2‐propanolate) in combination with water.[^21^, ^26^] Contrasting this, RE‐β‐diketonates often require strong oxidizing agents such as ozone (O_3_) as the already existing RE−O bonds, which contribute to the thermal stability, exhibit a low reactivity towards mild oxidizing agents. Furthermore, they require high volatilization temperatures of 140–170 °C in case of [Ce(thd)_3_]^[27]^ or [Yb(thd)_3_],^[28]^ (thd=2,2,6,6‐tetramethyl‐3,5‐heptanedionato), respectively which compromises on their applicability in the low temperature regime. RE‐cyclopentadienyl (RE‐Cp) complexes and their derivatives have demonstrated higher volatility in comparison to RE‐β‐diketonates while maintaining high thermal stability. The homoleptic precursor [YbCp_3_]^[20]^ was reported for thermal ALD of Yb_2_O_3_ with water as a co‐reactant and [CeCp_3_]^[29]^ was employed for CeO_2_ together with O_2_ plasma in a plasma‐enhanced ALD (PE‐ALD) process. Contrasting the former, the reactivity of the cerium derivate was insufficient to react with water and required a stronger oxidant. Addressing this crucial shortcoming, heteroleptic Ce complexes with Cp and amidinate ligands^[30]^ were introduced as ALD precursors. They possess higher thermal stability and volatility than the homoleptic RE‐Cp_3_ complexes and are reactive towards water. Furthermore, the precursors are liquid at room temperature and can be evaporated at 145 °C in case of the [CeCp_2_(*i*Pr‐AMD)] [bis‐isopropylcyclopentadienyl‐*N*,*N*′‐diisopropylacetamidinate‐cerium(III)]^[31]^ However, the preparation of these precursors is an intricate process and generally delivers low yields.

Homoleptic RE‐tris‐amidinates and tris‐guanidinates^[32]^ containing Gd, Dy, Er, Y have been demonstrated to be promising for the ALD of rare‐earth oxide (REO). In contrast to oxygen‐based ligands, the presence of six RE−N bonds makes them strongly oxyphilic, which promotes their high reactivity towards mild oxidizing agents like water. Moreover, steric, and electronic properties can be tuned by varying the steric bulk at the N‐C‐N backbone. In addition, the bidentate chelating effect of amidinates and guanidinates provides thermal stability to the resulting RE complex.^[24]^ Previously, RE‐guanidinates have been successfully utilized for the growth of RE oxides such as Gd_2_O_3_,^[33]^ Dy_2_O_3_,^[33]^ Er_2_O_3_ ^[34]^ and Y_2_O_3_ ^[35]^ in water‐assisted ALD. Furthermore, the reactivity of Er and Yb tris‐guanidinates was proven to be suitable enough for the deposition of inorganic–organic hybrid materials by atomic/molecular layer deposition (ALD/MLD).^[36]^ Apart from the experimental studies, the correlation of their thermal stability and their reactivity with different co‐reactants via theoretical calculations is advantageous to gain better insight into systematic precursor engineering, which to the best of our knowledge has not been carried out before for any rare earth precursors. The present study reports on all‐nitrogen coordinated RE complexes that are promising new Ce and Yb derivatives. Given that they are promising as highly reactive ALD precursors, a recent ALD study on the homoleptic amidinate [Ce(*N*‐*i*Pr‐AMD)_3_] (tris(*N*,*N*′‐diisopropylacetamidinato)cerium(III)) highlighted high evaporation temperatures of 170 °C.^[37]^

In order to address this and to understand the effect of the substituents on the N‐C‐N backbone of amidinates and guanidinates in terms of the physicochemical properties such as evaporation behavior, a series of Ce and Yb complexes was rationally designed. Herein, we report a systematic study by varying the organic moieties attached to the C atom of the N‐C‐N backbone by Me, NMe_2_, NEt_2_, and by varying the N substituent from *i*Pr to *t*Bu and Et to investigate the influence on the volatility, stability, and reactivity of a family of complexes.

Five different complexes of cerium and two different complexes of ytterbium, namely, tris(*N,N'*‐diisopropyl‐2‐dimethylamido‐guanidinato)cerium(III) [Ce(dpdmg)_3_] **1**, tris(*N,N'*‐butylethyl‐2‐dimethylamido‐guanidinato)cerium(III) [Ce(bedmg)_3_] **2**, tris(*N,N'*‐diisopropyl‐2‐diethylamido‐guanidinato)cerium(III) [Ce(dpdeg)_3_] **3**, tris(*N,N'*‐diisopropyl‐acetamidinato)cerium(III) [Ce(dpamd)_3_] **4**, tris(*N,N'*‐butylethyl‐acetamidinato)cerium(III) [Ce(beamd)_3_] **5**, tris(*N,N'*‐diisopropyl‐2‐dimethylamido‐guanidinato)ytterbium(III) [Yb(dpdmg)_3_] **6**, tris(*N,N'*‐diisopropyl‐acetamidinato)ytterbium(III) [Yb(dpamd)_3_] **7** were synthesized. The complex **7** is commercially available and patented.^[38]^

The complexes were structurally and thermally analyzed. An insight into the reactivity was additionally gained through first‐principles density functional theory (DFT) studies by a comparison of the structural changes and ligand loss energies of [Ce(dpdmg)_3_] **1**, [Ce(dpamd)_3_] **4**, [Yb(dpdmg)_3_] **6** and [Yb(dpamd)_3_] **7** precursors in contact with oxygen or a water molecule. Finally, a proof of principle ALD process was developed with the [Ce(dpdmg)_3_] **1** precursor, one of the promising cerium precursors of the series, using water as the co‐reactant. The resulting thin films were analyzed with respect to their crystallinity, composition, and optical properties.

## Results and Discussion

### Precursor synthesis and characterization

**Scheme 1:**
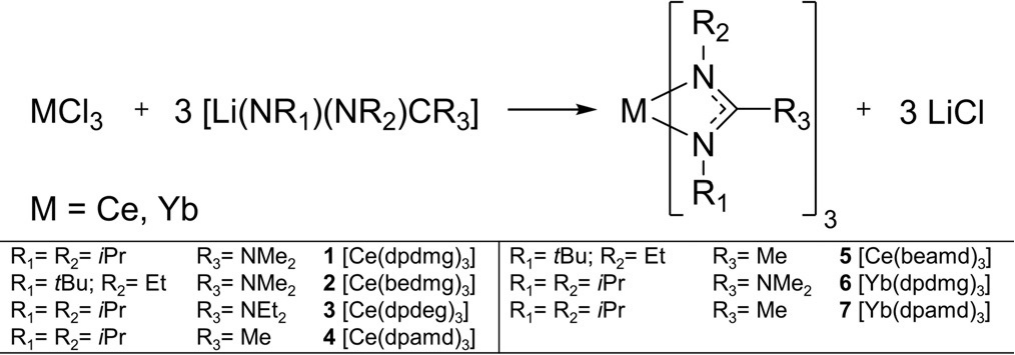
General reaction scheme for the synthesis of homoleptic rare‐earth guanidinate and amidinate complexes.

The synthesis of all the complexes **1**–**7** was achieved by a salt metathesis reaction of the anhydrous metal chloride MCl_3_ (M=Ce, Yb) and three equivalents of the respective lithium (Li) salts of the ligand [Li(NR_1_)(NR_2_)C(R_3_)] (R_1_=*i*Pr, *t*Bu; R_2_=*i*Pr, Et; R_3_=Me, NMe_2_, NEt_2_) as shown in Scheme 1.

The lithium salts were prepared in situ by insertion reaction of LiMe, LiNMe_2_, LiNEt_2_ into *N*,*N*′‐diisopropylcarbodiimide or *N*‐*tert*‐butyl‐*N*′‐ethylcarbodiimide in tetrahydrofuran (THF) for cerium complexes **1**–**5** and in diethyl ether (Et_2_O) for ytterbium complexes **6** and **7**. The purification of the products was achieved by recrystallization and/or sublimation. The products were obtained in good yields (70–90 %). From the handling of the complexes during synthesis, some general statements can be derived. All the complexes have good solubility in THF, pentane, hexane, benzene, Et_2_O and can be applied as potential precursors for chemical solution deposition thin film processes as well. The cerium complexes **1**–**5** are extremely sensitive to air and moisture and immediately turn black when they are exposed to air and moisture; these observations are in agreement with those for other organo‐cerium complexes.^[39]^

**Figure 1:**
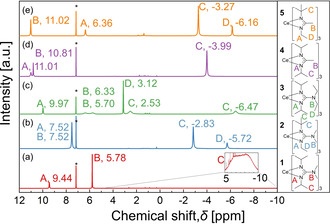
^1^H NMR spectra of the cerium complexes **1**–**5** in C_6_D_6._ (*C_6_D_6_, 7.16 ppm).

All the complexes are paramagnetic in nature (one unpaired electron in Ce^3+^ and one in Yb^3+^), necessitating large chemical shift ranges (ppm) for the measurement of proton and carbon nuclear magnetic resonance (NMR) spectra of the cerium complexes. For the ytterbium complexes, the recording of the spectra failed due to significant paramagnetic shifts. ^1^H NMR was measured for compounds **1**–**5** in C_6_D_6_, the chemical shift peaks were found to be broad in the range −10 ppm to 11 ppm and the intensity was significantly reduced. However, the integration of the peaks matched with the number of hydrogen atoms present in the complexes (Figure 1). A detailed NMR study was done to relate the paramagnetic shifts caused due to the unpaired electron of Ce^3+^ in the complexes. ^13^C NMR was performed (Figure S1 in the Supporting Information) and correlated with the ^1^H NMR by ^1^H–^13^C heteronuclear single quantum coherence (HSQC) measurements (Figures S2, S4, S5) to identify a relation between the carbons bound to the protons. All the details are provided in the Supporting Information.

The ^1^H NMR (Figure 1) shows that for the guanidinate complexes **1** and **2** the peak B corresponding to the protons of the methyl group in the C‐NMe_2_ moiety is at a chemical shift of 5.78 ppm (Figure 1 a) and 7.52 ppm (Figure 1 b), respectively. For the complex **3** (Figure 1 c), chemically inequivalent CH_2_ protons for the C‐NEt_2_ moiety are observed at a chemical shift of 6.33 ppm and 5.70 ppm (peak B) whereas for the CH_3_ protons of the C‐NEt_2_ moiety a signal at 3.12 ppm (peak D) can be seen. For the amidinate complexes **4** and **5**, the signal of the CH_3_ protons of the C‐Me moiety (peak B) is at 10.81 ppm (Figure 1 d) and 11.02 ppm (Figure 1 e) respectively. The chemical shifts of the protons of the CH_2_ group in the Et moiety for the asymmetrical guanidinate and amidinate complexes **2** (Figure 1 b) and **5** (Figure 1 e) can be found (peak A) at 7.52 ppm and 6.36 ppm, the CH_3_ protons of the Et group (peak D) at −5.72 ppm and −6.16 ppm and the CH_3_ protons of the *t*Bu groups (peak C) at −2.83 ppm and −3.27 ppm, respectively. Noticeably, the CH_3_ protons of the *i*Pr moieties are similar for the complexes **1**, **3**, and **4** (peak C) but behave very differently in our NMR studies: While the peak C in Figure 1 for complex **1** is very broad in the range of −10 ppm to 4 ppm (Figure 1 a), for complex **3** it is split into two peaks at 2.53 ppm and −6.47 ppm, (Figure 1 c) and for complex **4** it is a single sharp peak at −3.99 ppm. (Figure 1 d) To identify the reason, temperature dependent ^1^H NMR was performed on complex **1**, Figure S3. From the spectra, at −50 °C two clear peaks at 4.33 ppm and 10.30 ppm for the CH_3_ protons of the *i*Pr group are observed. This can be explained by a hindered rotation of the CH_3_ protons, resulting in chemically different environments for the CH_3_ groups and hence, splitting of the signal is observed. As the temperature increases, these two peaks are broadened and at 50 °C, both the peaks coalesce to form one peak at 1.91 ppm. Upon further heating to 100 °C, it forms a sharp peak at 1.50 ppm. At higher temperatures, the rotation of the ligand is increased and hence the CH_3_ moieties of the *i*Pr groups rotate fast enough that the CH_3_ protons become equivalent yielding one signal. Similarly, for complex **3** even higher steric hindrance caused by the NEt_2_ group restricts the rotation for the CH_3_ groups of the *i*Pr moieties and facilitates the appearance of two peaks (peak C) at 2.53 ppm and −6.47 ppm at room temperature. In complex **4**, the steric hindrance is the lowest due to the small Me group at the N‐C‐N backbone and hence the rotation is not hindered, which leads to one signal at −3.99 ppm at room temperature. Furthermore, for complex **1** in Figure S3, the CH protons of the *i*Pr group (peak A) and the CH_3_ protons of C‐NMe_2_ group (peak B) are shifted upfield with increasing temperature because the binding strength of the ligand lowers due to the increased rotation and hence electron density increases at the ligand.

Electron‐impact mass spectrometry (EI‐MS) analysis was carried out to confirm the formation of the target compound, analyze the fragmentation pattern, and to get an insight into the structural features of the metal‐organic complexes. The EI‐MS spectra for all the complexes are given in the Supporting Information (Figures S6–S12) and selected peaks with assigned fragments are listed in Table 1. For all the complexes, the respective molecular ion peaks (ML_3_^+^) with expected mass to charge ratios (*m*/*z*) and considerable relative intensities of 14.8 % for **1**, 9.4 % for **2**, 46.8 % for **3**, 38.8 % for **4**, 44.6 % for **5**, 2.0 % for **6**, 7.0 % for **7** were found. Peaks at higher *m*/*z* ratios than the molecular ion peak were not observed under experimental conditions, which suggests that all the complexes can exist as monomers in the gas phase. For all the complexes, the fragments from cleavage of one ligand (ML_2_^+^), or of two ligands (ML^+^) species, as well as the ligand L^+^ itself, were detected. Interestingly, for all amidinate complexes, the fragment with the highest intensity (100 %) is the ML_2_^+^ fragment. The guanidinates seem to decompose into smaller fragments, indicated by the peak with 100 % intensity which is observed for fragments associated with the organic ligands and their decomposition fragments. A similar fragmentation behavior is observed for the literature known rare‐earth tris guanidinates[^24^, ^40^] and tris‐amidinates[^40a^, ^41^]complexes.

**Table 1:** Overview of m/*z* (relative intensity %) of selected possible fragments observed for complexes **1**–**7** detected from EI‐MS.

Fragments	[Ce(dpdmg)_3_] **1**	[Ce(bedmg)_3_] **2**	[Ce(dpdeg)_3_] **3**	[Ce(dpamd)_3_] **4**	[Ce(beamd)_3_] **5**	[Yb(dpdmg)_3_] **6**	[Yb(dpamd)_3_] **7**
ML_3_^+^	650.5 (14.8 %)	650.5 (9.4 %)	734.5 (46.8 %)	563.4 (38.8 %)	563.4 (44.6 %)	684.8 (2.0 %)	597.5 (7.0 %)
ML_2_^+^	480.3 (35.4 %)	480.3 (13.7 %)	536.3 (40.8 %)	422.2 (100 %)	422.2 (100 %)	513.4 (92.5 %)	455.4 (100 %)
ML^+^	311.4 (13.2 %)	311.4 (15.9 %)	337.1 (49.4 %)	280.1 (3.4 %)	280.1 (5.9 %)	342.2 (94.2 %)	313.2 (28.9 %)
ML^+^‐(*i*Pr/*t*Bu)	267.1 (6.0 %)	253.1 (9.0 %)	–	–	–	301.1 (36.2 %)	–
ML^+^‐(2CH_2_CH_2_)	–	–	282.1 (48.4 %)	–	–	–	–
L^+^	171.2 (5.8 %)	171.2 (1.8 %)	199.3 (5.8 %)	142.2 (17.5 %)	142.2 (18.8 %)	170.2 (25.0 %)	142.2 (28.6 %)
*i*PrNC^+^	69.1 (100 %)	–	69.1 (91.0 %)	–	–	69.1 (100 %)	–
EtNC^+^	–	57.0 (100 %)	–	–	–	–	–
*t*BuNC^+^	–	83.0 (60.3 %)	–	–	–	–	–
*i*Pr^+^	43.1 (75.6 %)	43.1 (19.6 %)	43.1 (100 %)	43.1 (10.1 %)	43.1 (10.2 %)	43.1 (94.3 %)	43.1 (21.8 %)

**Figure 2:**
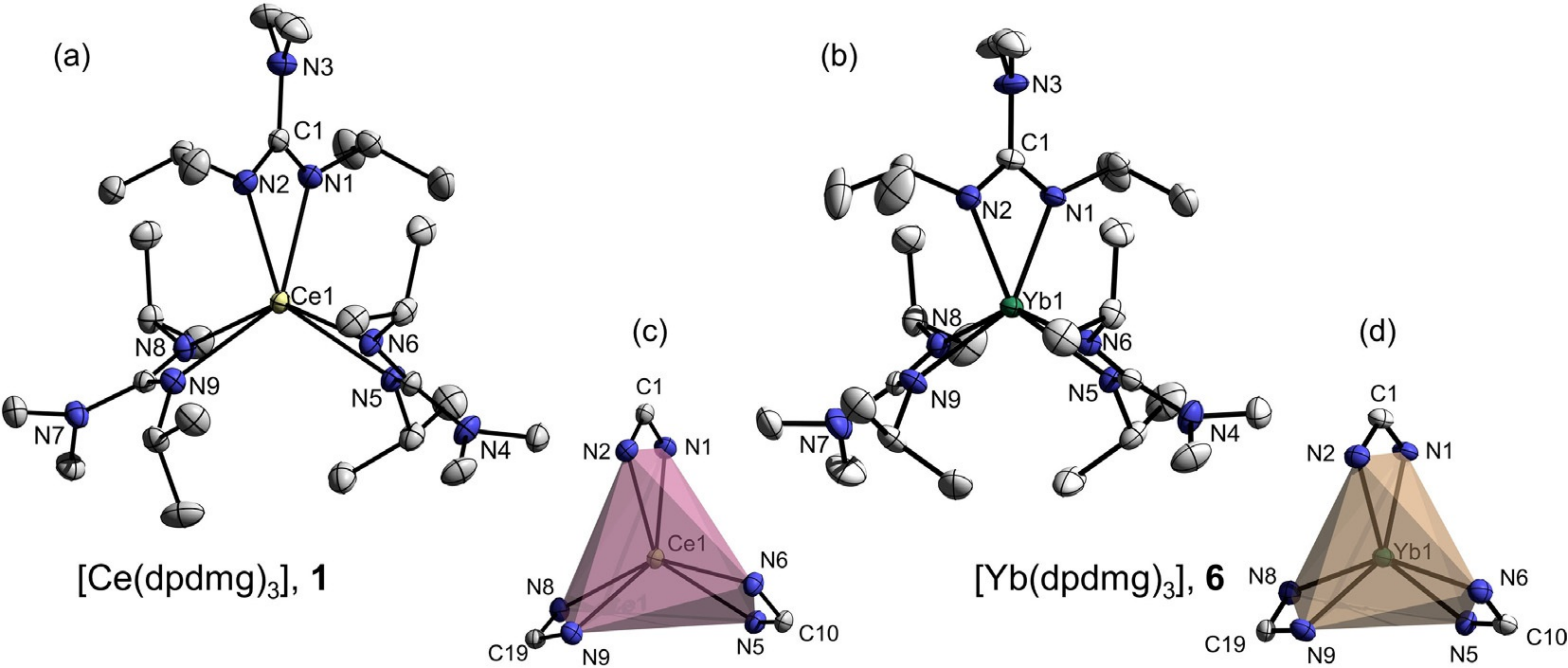
Molecular solid‐state structure (a)–(b) and coordination polyhedron (c)–(d) of [Ce(dpdmg)_3_], **1** and [Yb(dpdmg)_3_], **6** with 50 % thermal ellipsoid probability. Hydrogen atoms are omitted for clarity.

The molecular structures of **1** and **6** were determined by single‐crystal X‐ray diffraction (SC‐XRD) and are depicted in Figure 2 a,b while the crystallographic data is given in Table S1. The crystals of suitable quality for measurement could not be obtained for other complexes. Complex **1** crystallizes in the monoclinic crystal system in the *C*2/*c* space group with 8 molecules per unit cell having a calculated density of 1.299 g cm^−3^. Complex **6** crystallizes in the triclinic crystal system in the *P* $\bar{1}$ space group with two molecules per unit cell having a density of 1.299 g cm^−3^. Both the complexes exist as monomers in the solid‐state and are isostructural with six‐fold coordination of nitrogen to the lanthanide center. Thus, it is surrounded by three bidentate η^2^‐guanidinato ligands with a trigonal planar structure. This structure is accounted to a delocalized π‐electron system as known from the literature^[24]^ and indicated by a mean bond length of N_b_−C=1.336(5) Å for **1** and 1.335(1) Å for **6**, and N_m_−C=1.400(3) Å for **1** and 1.399(4) Å for **6** (Table 2), that is shorter than a typical C−N single bond length of 1.474 Å.^[42]^ Here, the nitrogen atoms which constitute the N‐C‐N backbone are labeled as N_b_ (N1/N2/N5/N6/N8/N9) and the nitrogen atoms connected to methyl groups are labeled as N_m_ (N3/N4/N7).

**Table 2:** Selected bond lengths, bond angles, bite angles and torsion angles of the synthesized complexes [Ce(dpdmg)_3_] **1** and [Yb(dpdmg)_3_] **6** with N_b_=N1/N2/N5/N6/N8/N9; N_m_=N3/N4/N7; Q1 is the centroid of N1, N5, N8 and Q2 is the centroid of N2, N6, N9.

	[Ce(dpdmg)_3_] **1**	[Yb(dpdmg)_3_] **6**
Bond length [Å]		
M−N1	2.510(9)	2.341(2)
M−N2	2.486(8)	2.341(3)
M−N5	2.514(1)	2.329(3)
M−N6	2.480(3)	2.336(2)
M−N8	2.527(4)	2.327(2)
M−N9	2.481(1)	2.338(2)
mean M−N_b_	2.500(1)	2.335(6)
mean C−N_b_	1.336(5)	1.335(1)
mean C−N_m_	1.400(3)	1.399(4)

Bite angle [°]		
N1‐M‐N2	53.92(6)	57.61(9)
N5‐M‐N6	53.94(6)	57.78(9)
N8‐M‐N9	53.48(6)	57.74(8)
mean N_b_‐M‐N_b_	53.78(6)	57.71(9)

Bond angle [°]		
N2‐M‐N6	98.38(6)	101.69(9)
N6‐M‐N9	101.84(6)	101.37(9)
N9‐M‐N2	102.52(6)	101.32(9)
N1‐M‐N5	105.48(6)	101.29(9)
N5‐M‐N8	105.51(6)	99.76(9)
N8‐M‐N1	102.38(6)	102.04(9)
		
Torsion angle [°]		
N1‐Q1‐Q2‐N2	15.38	22.45
N5‐Q1‐Q2‐N6	17.65	22.61
N8‐Q1‐Q2‐N9	17.90	21.62
mean N_b_‐Q1‐Q2‐N_b_	16.98	22.23

**Figure 3:**
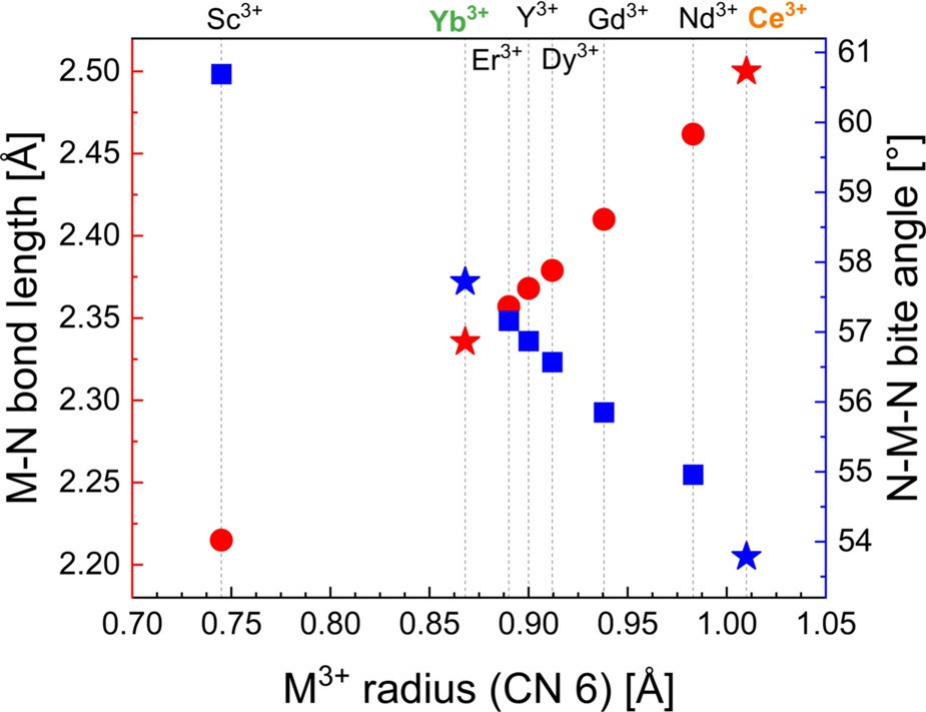
Relation of bond length of M‐N, bite angle *N*‐M‐N, and ionic radii of M^3+^ having coordination number (CN) 6 for isostructural homoleptic [M(dpdmg)_3_]. Red‐colored data points represent the M−N bond length vs. the ionic radii of M^3+^ and blue‐colored data points represent the N‐M‐N bite angle vs. the ionic radii of M^3+^. This graph has been adapted and modified from Milanov et al.^[40b]^

The coordination geometry of the complexes (as shown in Figure 2 c and d) can be described as distorted trigonal prismatic. This is indicated by the torsion angle N_b_‐Q1‐Q2‐N_b_, ranging between 15.38° and 17.90° for **1** and between 21.62° and 22.61° for **2** (Table 2), where Q1 is the centroid of the backplane of the prism spanned by N1, N5, N8, Q2 is the centroid of the front plane of the prism spanned by N2, N6, N9 and here, N_b_ are the nitrogen atoms of the same guanidinate ligand that are coordinated to the metal center. Ideally, the torsion angle is 0° for a trigonal prismatic geometry and 60° for the octahedral geometry. Thus, the structure is distorted by a twisted offset of the two planes toward each other. Furthermore, in complex **1**, the N‐M‐N bond angles (Table 2) range between 102.38(6)° and 105.51(6)° for the plane spanned by N1, N5, N8, and between 98.38(6)° and 102.52(6)° for the plane spanned by N2, N6, N9. For complex **2** the situation is similar with N‐M‐N angles ranging from 99.76(9)° to 102.04(9)° within the N1, N5, N8 plane and from 101.32(9)° to 101.69(9)° within the N2, N6, N9 plane. Here, the bond angles are similar because the planes are connected by the N‐C‐N backbone which hinders distortions in the distance of the planes to the metal center. The planes containing N1, N5, N8 and N2, N6, N9 are nearly parallel having a dihedral angle of 1.135° for **1** and 0.361° for **6**. The bond lengths and bite angles of the guanidinate ligands to the metal center as well as their mean values for both the complexes, are additionally listed in Table 2 and can be correlated to other lanthanide guanidinate complexes, reported earlier (Figure 3).

The previously reported isostructural complexes bearing identical guanidinate ligands[^24^, ^40^] for rare earth metals show a relationship between their effective ionic radius (M^3+^)^[43]^ and specific geometrical parameters including the M−N bond length and the N‐M‐N bite angle of the guanidinate ligand. The ionic radius along the series of lanthanides (La–Lu) decreases due to poor shielding of the 4f electrons known as lanthanide contraction.^[44]^ The ionic radius for Ce^3+^ and Yb^3+^ is 1.01 Å and 0.868 Å, respectively.^[43]^ As can be seen from Table 2 the M−N bond length in compounds **1** and **6** range from 2.480(3) Å to 2.527(4) Å for **1** and from 2.327(2) Å to 2.341(3) Å for **6**. The different M−N bond lengths result in mean bond lengths of 2.500(1) Å for **1** and 2.335(6) Å for **6** which is in agreement with the trend depicted in Figure 3 of a longer M−N bond for an increasing ionic radius. The N‐M‐N bite angles of the guanidinate ligands are ranging between 53.48(6)° and 53.94(6)° for **1** and 57.61(9)° and 57.78(9)° for **6** which results in a mean bite angle of 53.78(6)° for **1** and 57.71(9)° for **6**. This observation is matching again the trend depicted in Figure 3, indicating a smaller bite angle for larger rare‐earth ion centers.

As the guanidinate ligand itself is quite rigid because of π system, the trends in the M−N bond lengths and N‐M‐N bite angles results in the twist of the trigonal planes of the trigonal prismatic structure, expressed by the N_b_‐Q1‐Q2‐N_b_ torsion angle. This is larger for a smaller rare earth ion. The mean values are 16.98° for **1** and 22.23° for **6** which match this trend.

### Evaluation of thermal properties

To evaluate the potential application of a compound as a precursor for ALD applications, the study of the thermal properties is important and in this context the volatility, melting point, and thermal stability were investigated.

With low melting points being generally desirable, they can also be used as a first indicator for the extent of intermolecular interactions present in a compound. The melting point of the metal‐organic complexes was analyzed by differential scanning calorimetry (DSC, not shown), and the results are summarized in Table S2. It is found that the melting point for complex **1** is 104 °C and for complex **2** the melting point is 88 °C. The difference can be explained by the asymmetry in the molecule because of which the crystal packing and hence the entropy of crystallization is affected.^[45]^ Complex **3** has a higher melting point of 134 °C. The lowest melting point of 50 °C is obtained for complex **5** which can be ascribed to a high asymmetry in the molecule. The ytterbium complex **6** is melting at a higher temperature of 110 °C than its isostructural cerium analog **1**. No melting point was observed for the complexes **4** and **7**.

**Figure 4:**
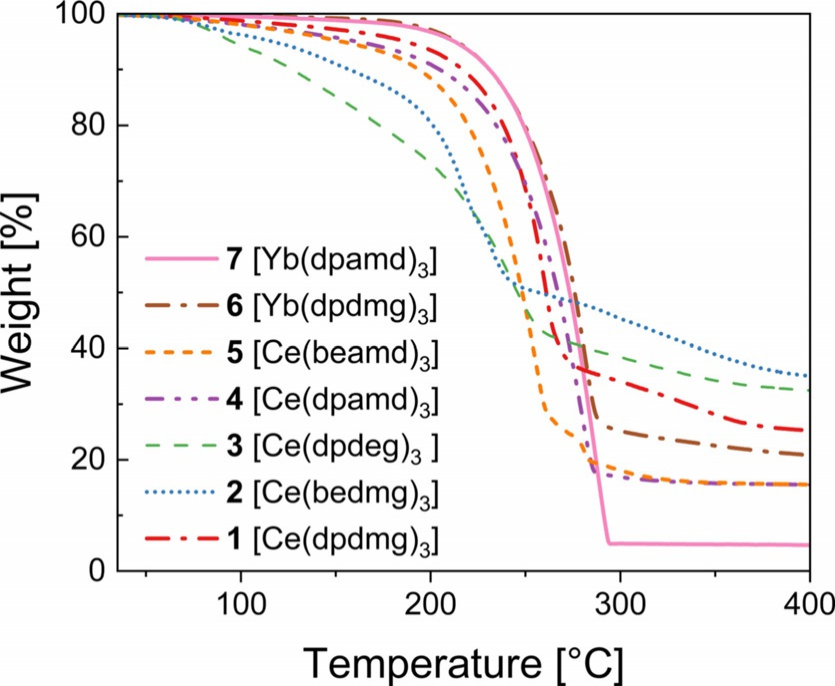
Thermogravimetric analysis of complexes **1**–**7**.

Thermogravimetric analysis (TGA) was employed to study the evaporation behavior and stability of complexes **1**–**7**. The weight loss of the complexes as a function of temperature in the range of 35 °C−400 °C is shown in Figure 4, and their onset of volatilization [°C] and residual weight [%] are given in Table S2.

As it can be seen for the Yb complex **7**, the initial mass loss is very low and the onset of volatilization, here defined as the temperature at which 1 % weight loss occurs, is at 110.6 °C. The main weight loss step is observed at a higher temperature of 253.5 °C (here defined as the step temperature assessed by the method of tangents^[46]^) after which a residual weight of 5.2 % was observed. Thus, the weight loss can mostly be attributed to the evaporation of the intact complex. Complex **6** shows an onset of volatilization at 155.3 °C which is significantly higher than that of compound **7**, and the step temperature is 253.3 °C which is nearly the same temperature as for complex **7**. The residual mass of **6** is 20.8 % which is considerably higher than that of **7**, yet lower than any expected decomposition product of Yb (nitride, carbide). Hence, it indicates that thermal evaporation overlaps with decomposition from which evaporation is the predominant phenomenon in both Yb complexes. However, the volatility of **7** is higher than that of **6** as indicated by a lower onset temperature of volatilization for **7** and a lower rest mass. The potential applicability of the complexes **6** and **7** is indicated based on the TGA of the Yb complexes.

Observations on the Ce compounds appear to be partly contrasting. While the residual weights of all TG measurements for the complexes **1**–**5** were not negligible, they are still lower than possible Ce decomposition products (nitrides, carbides), indicating again the coexistence of evaporation and decomposition under the applied experimental conditions upon increased heat exposure. Complex **4** shows a one‐step weight loss with an onset of volatilization at 70.8 °C, and the step temperature is 239.9 °C. For the asymmetric complex **5**, the onset temperature is slightly higher with 76.4 °C than for **4** and a two‐step weight loss is observed. The first step at a temperature of 218.5 °C is the major step which ends with a rest mass of 27.5 %. However, the remaining substance, likely a decomposition product, undergoes further evaporation at a second step at 277.8 °C resulting in a final residual mass of 15.5 %. Hence, the asymmetry in complex **5** results in lower thermal stability compared to complex **4**. For complex **1**, the onset is 90.1 °C which is higher than for the homoleptic amidinates of cerium, but the step temperature of 239.6 °C is similar for **1** and **4**. This indicates that the complex **4** is more volatile than complex **1**. After this step, further weight loss can be observed before it becomes constant with a residual weight of 25.2 % indicating that the decomposition product is still volatile. The complexes **2** and **3** show onset temperatures of 76.4 °C and 67.1 °C, respectively. The step temperature for **2** is at 193.1 °C and for **3** at 197.3 °C and is again accompanied by decomposition. The residual masses obtained were 34.1 % and 33.4 %, respectively, indicating lower thermal stability for the complexes **2** and **3** when compared to complex **1**.

This study exemplifies how systematic variation of substituents on the side chains and backbones of the employed amidinate and guanidinate ligands can be used to tune the thermal properties of the precursors. The thermal properties of complexes having the same molecular mass and an identical composition, namely **1**, **2**, and **4**, **5**, differ noticeably. Due to the large asymmetry in its structure, complex **5** has the lowest melting of all the complexes and complex **2** possesses a lower melting point than **1**. On the other hand, the higher asymmetry in the molecular structure can lead to lower thermal stability and it was additionally found that the thermal stability and volatility of the amidinates is higher than the one of the guanidinates. Interestingly, the cerium guanidinate **1** is melting at a lower temperature than the isostructural ytterbium complex **6**. Besides, the cerium complexes **1**–**5** are found to be more volatile than ytterbium complexes **6** and **7** while exhibiting less thermal resilience.

### DFT studies

To obtain an insight into the fundamental aspects of the chemistry of the compounds on the molecular level, DFT was used to model the atomic structures and to simulate the reactivity of the complexes towards potential co‐reactants. In the first set of calculations, the precursors [Ce(dpdmg)_3_] **1**, [Ce(dpamd)_3_] **4**, [Yb(dpdmg)_3_] **6** and [Yb(dpamd)_3_] **7** were modeled as isolated molecules in vacuum at zero Kelvin (K) and zero Giga Pascal (GPa), with the relaxed atomic structures shown in Figure S13. The geometries of the complexes were accurately reproduced by DFT calculations. The M−N bond lengths and bite angles (N‐M‐N) of the optimized structures **1**, **4**, **6**, and **7** are given in Table S3 and Table S4 respectively and are consistent with the solid‐state studies.

The bond dissociation energy defined as the energy for the removal of the first ligand, was computed for all compounds. Based on bond dissociation energies, as shown in Table 3, it is anticipated that cerium guanidinate **1** will be more stable compared to cerium amidinate **4** under vacuum conditions. It is to be noted that, under thermal conditions other reactions or decomposition pathways can also occur; for example, for guanidinates, the carbodiimide deinsertion^[47]^ can also take place which has not been taken into consideration for DFT experiments. For the Yb precursors, the trend is the same, although the difference is less significant. Taking into consideration the bond dissociation energies, **6** and **7** would have similar stability under vacuum conditions.

**Table 3:** Computed bond dissociation energies to lose the first ligand from the precursors *E* ^ligand^ [kJ mol^−1^] in vacuum and in models of interaction with oxygen and water.

	In vacuum	In the presence of a O_2_ molecule	In the presence of a H_2_O molecule
	*E* ^ligand^ [kJ mol^−1^]	*E* ^ligand^ [kJ mol^−1^]	*E* ^ligand^ [kJ mol^M‐>1^]
**1** [Ce(dpdmg)_3_]	626.37	69.02	85.92
**4** [Ce(dpamd)_3_]	405.13	85.38	104.20
**6** [Yb(dpdmg)_3_]	373.25	332.74	68.56
**7** [Yb(dpamd)_3_]	352.40	336.29	90.73

Based on the bond dissociation energies and the overall trend described above, the stability and potential reactivity of these precursors is not necessarily correlated to the bonding properties within their molecular structure. We would expect that precursors with the shorter M−N bonds would be less reactive; however, this is not apparent from the data in Table S3 and Table 3.

**Figure 5:**
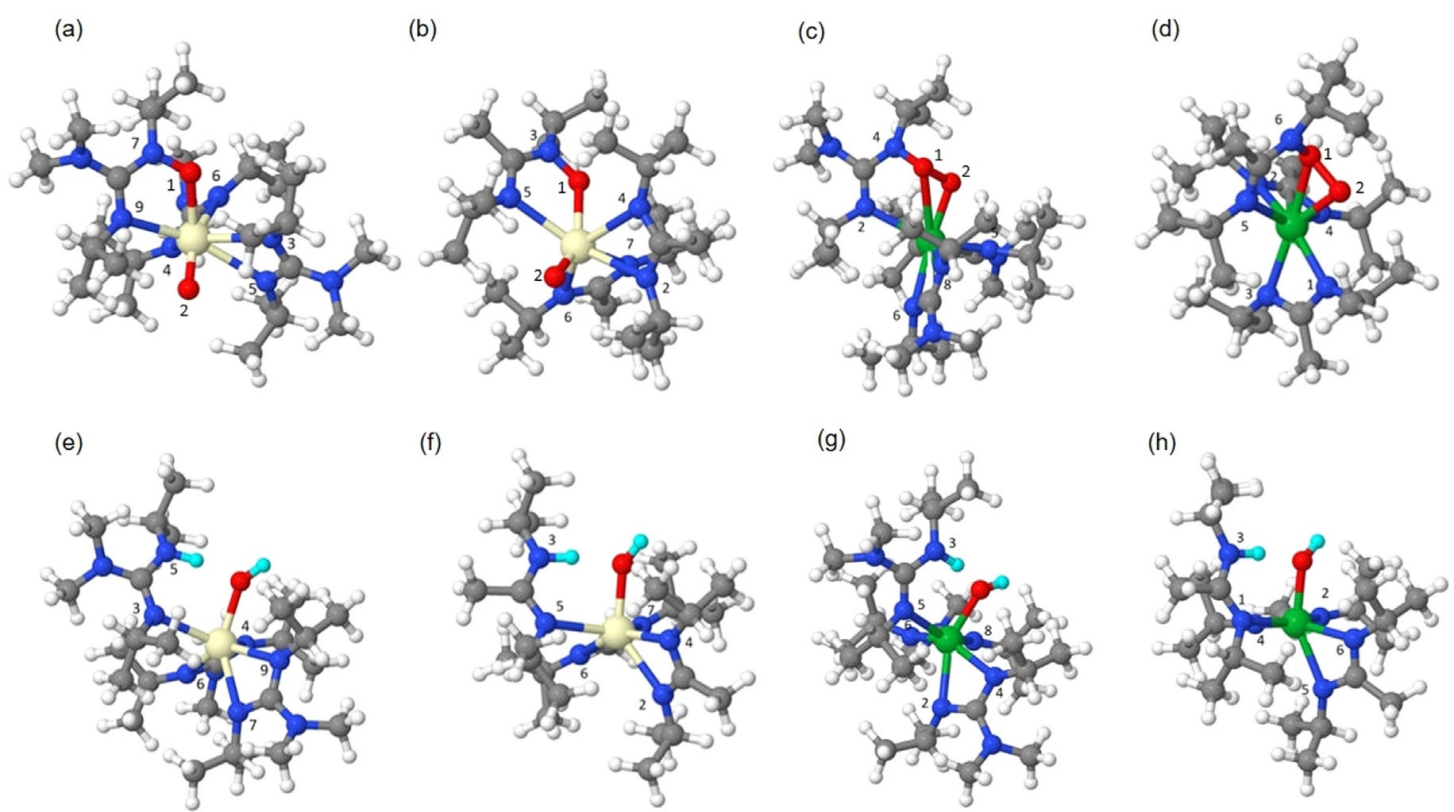
Atomistic structures of (a) [Ce(dpdmg)_3_] **1**, (b) [Ce(dpamd)_3_] **4**, (c) [Yb(dpdmg)_3_] **6** and (d) [Yb(dpamd)_3_] **7** after incorporating one oxygen molecule, (e) [Ce(dpdmg)_3_] **1**, (f) [Ce(dpamd)_3_] **4**, (g) [Yb(dpdmg)_3_] **6** and (h) [Yb(dpamd)_3_] **7** after incorporating one water molecule. The color coding is as follows Cream: Cerium, Green: Ytterbium, Blue: Nitrogen, Gray: Carbon, White: Hydrogen. Red: Oxygen. Cyan H from water. All the numbers labelled on Nitrogen atoms are according to Table S5 and Table S6.

Keeping this in mind, we expanded the model system of the precursors to include the interaction with one O_2_ and one H_2_O molecule, respectively and investigated the reactivity again at zero K and zero GPa. An O_2_ molecule was placed at 2.50 Å from the metal center in its gas phase geometry and was allowed to relax. Figure 5 a–d shows the relaxed atomic structure of the precursors after the incorporation of the O_2_ molecule and demonstrates that the interaction with O_2_ depends on the metal center. Ce promotes the breaking of the O=O bond in both precursors, which is typical for Ce^3+^ species.^[48]^ For the complexes **1** and **4** (Figure 5 a,b) one oxygen atom inserts into the original Ce−N bond creating new Ce−O and O−N bonds while the second oxygen atom binds with the Ce center for both the complexes forming an oxo‐ligand.

In contrast to cerium, ytterbium does not break the O=O bond, Figure 5 c,d. Instead, in the complexes **6** and **7**, the O_2_ molecule forces its way to close proximity to the metal center and forms a tricycle with Yb while one Yb−N bond is cleaved, which again results in a 7‐fold coordination sphere. One of the oxygen atoms forms an O−N bond with the non‐metal coordinated N. The O−O bond length is found to be in the range of 1.45 Å to 1.46 Å which is characteristic of a peroxide species. Table S5 shows the M−O, O−N, and M−N bonds in the presence of oxygen.

According to bond dissociation energies shown in Table 3, **1** would be more reactive compared to **4** with O_2_. For the Yb precursors, the difference in bond dissociation energies is almost negligible and slightly changed from the gas phase precursor. This suggests that the reactivity of the ytterbium containing precursors is little affected by the nature of the ligand regarding the interaction with O_2_.

Figure 5 e–h shows the optimized structures of the precursors after the interaction with one H_2_O molecule. When one H_2_O molecule interacts with the precursors, it preferably binds to the central Ce and Yb atom and dissociates. The OH group of water binds to the M, and the remaining H atom binds to nitrogen upon metal‐nitrogen bond breakage.

Table S6 shows the M−OH and M−N bonds in the presence of water. Once the water molecule has reacted with the metal center, dissociation of the semi‐protonated, solely one‐fold bonded ligand was identified as a preferential dissociation pathway. Based on the computed bond dissociation energies, **1** is expected to be more reactive compared to **4**. and **6** would be more reactive compared to **7**. Thus, the reactivity of all these precursors can be strongly influenced by the interaction with O_2_ or H_2_O molecules. This study shows that these precursors are potential candidates for ALD precursors. Interestingly, the bond dissociation energy for the cerium complexes **1** and **4** is found to be less in the presence of an oxygen molecule than in the vicinity of a H_2_O molecule which suggests that the elemental O_2_ could be an interesting co‐reagent for ALD with Ce complexes.

### ALD of CeO_2_ thin films

Based on the promising results obtained from the thermal characterization of the precursors as well as the DFT studies, the next objective was to evaluate the precursors for ALD applications. As a representative case, we chose [Ce(dpdmg)_3_] **1** as it was found to be very reactive towards water based on our DFT calculations. There are very few reports on water assisted ALD, as highlighted in the introduction. Thus, such a study can widen the library of water assisted ALD processes for RE oxides. In this context, proof‐of‐principle ALD experiments on Si(100) substrates were performed with [Ce(dpdmg)_3_] **1** using water as co‐reactant.

**Figure 6:**
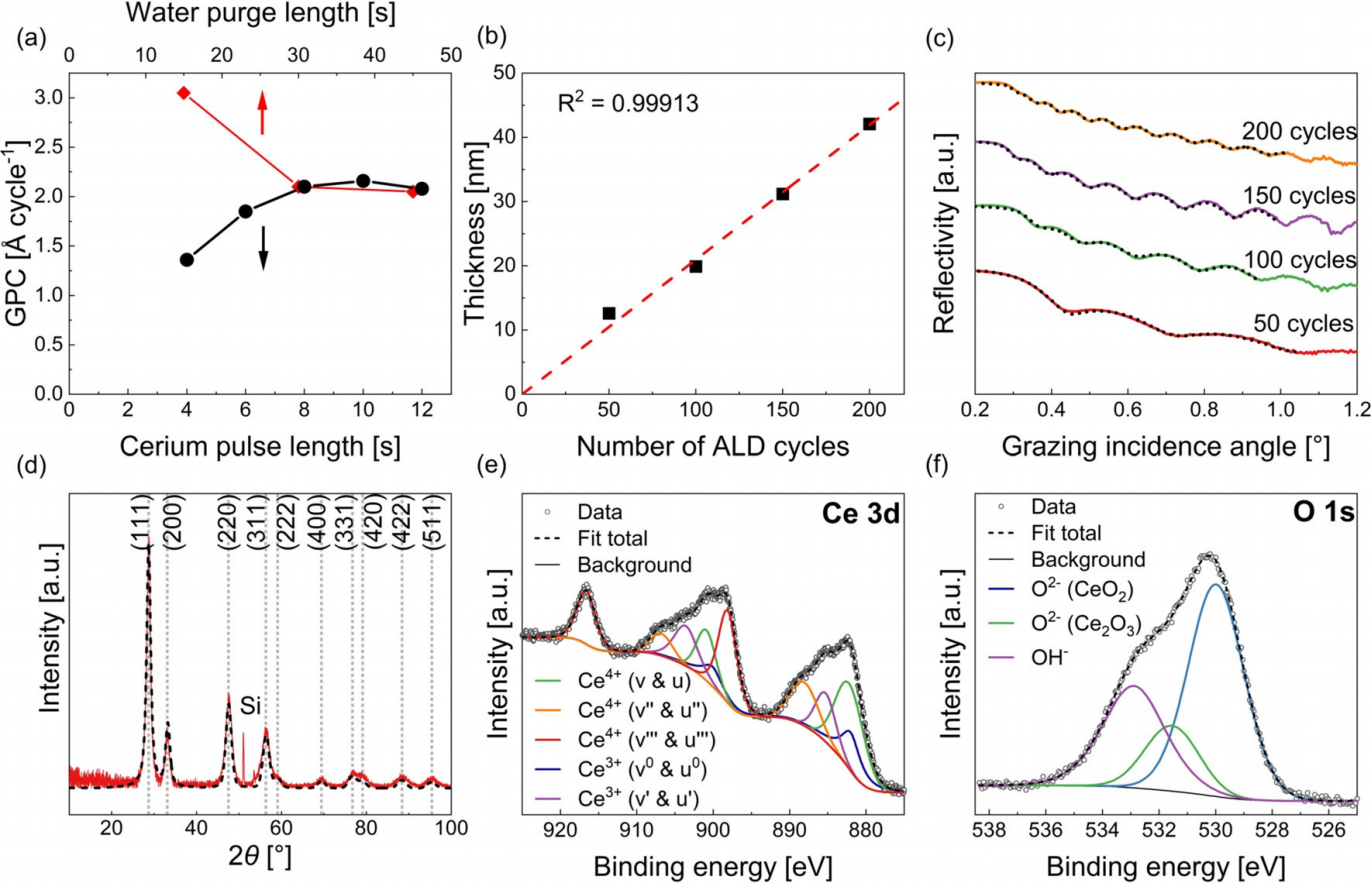
(a) The black data points represent precursor saturation studies by varying [Ce(dpdmg)_3_] **1** pulse length and the red data points represent the variation of GPC with water purge time (b) Thickness of the film vs. number of applied ALD cycles; both at the deposition temperature of 160 °C on Si(100). (c) XRR patterns of films with varying total number of cycles (black dotted line represents the simulated pattern) (d) GIXRD patterns at an incident angle of 0.5° of the film deposited on Si (the black dashed line represents the refined computational pattern with reference to cubic CeO_2_ (ICDD: 04–016–4620)) (e) XPS analysis of the Ce 3d core level spectrum of the as introduced surface of a 42 nm thick CeO_2_ film grown on Si(100). Experimental and fitting curves for all spin‐orbital splitting's are given following the nomenclature of Preisler et al. ^[49]^ (f) XPS analysis of the O 1s core level spectrum for the same film. Experimental and fitting curves for all oxygen components are given.

To verify the self‐limiting nature of the thin film growth, a saturation study of the precursor vaporized at 140 °C and the co‐reactant water maintained at room temperature was carried out for a deposition temperature of 160 °C (Figure 6 a). The precursor pulse length was varied from four to twelve seconds, while the other parameters were kept constant with a precursor purging time of 30 s, a water pulse length of 3 s, and water purging time of 30 s. As seen in Figure 6 a, the precursor saturates after 8 s pulse with a constant GPC of 2.1 Å, thereby confirming a self‐limiting growth. Similarly, the water purge length was varied from 15 s to 45 s, Figure 6 a, while the other parameters were kept constant. There was no change in the GPC after more than 30 s of water purge. The increased growth was observed below 30 s of water purge time, probably due to a reaction of additional adsorbed water molecules on the surface with the precursor, which has also been observed in a similar process for the deposition of Y_2_O_3_ in the same reactor type.^[35]^ The dependency of the film thickness on the number of applied cycles was subsequently analyzed as shown in Figure 6 b for the precursor **1** pulse/purge/water pulse/purge sequence of 8 s/30 s/3 s/30 s (illustrated in Figure S14). The obtained fit value *R* ^2^ of 0.99913 shows that for each cycle, the same amount of material is deposited and therefore, the thickness can be tuned precisely. These initial set of results in terms of validating ALD growth characteristics further confirm that the precursor is suitable for water assisted ALD. More detailed experiments varying the process parameters have to be performed to optimize the new ALD process for CeO_2_.

The crystallinity of ALD grown thin films was assessed by grazing incidence X‐ray diffraction (GIXRD). As exemplarily illustrated by the GIXRD pattern obtained for a 42 nm thick film grown at 160 °C (Figure 6 d) the as‐deposited layers possess a polycrystalline nature matching the computed reflections of a refined cubic CeO_2_ reference pattern (ICDD: 04–016–4620). X‐ray reflectivity (XRR) patterns obtained for films with varying total number of cycles, i.e., different thicknesses, are shown in Figure 6 c. From the respective fits, an average thin film density of around 5.0 g cm^−3^ could be estimated based on the critical angle fitting.

Seeking further evidence for the formation of the high‐valent CeO_2_ phase and to obtain insights into the chemical composition of the films, X‐ray photoelectron spectroscopy (XPS) was conducted on a 42 nm thick film deposited on Si(100) substrate. The Ce 3d core level spectrum of the as introduced film is shown in Figure 6 e and represents the conditions of the surface in a maximum depth of around 5 nm. Contributions of both Ce^4+^ and Ce^3+^ species at the expected binding energies (listed in the Supporting Information, Table S7) to the core level were identified as the formation of oxygen vacancies and partial reduction of Ce^4+^ to Ce^3+^ species are known phenomena on ceria surfaces.^[50]^ Following the method developed by Romeo et al.^[51]^ that is well described by Preisler et al.,^[49]^ fitting of all spin‐orbital and splitting components and summation of peak areas associated to Ce^3+^ and Ce^4+^ (see Table 4) allowed to estimate the concentration of the first‐mentioned to be 24.8 % and of the latter‐mentioned to be 75.2 %. Hereby the components described as v^0^ and u^0^ as well as v“ and u” represent Ce^3+^ species while v, u, v′′, u′′ as well as v′′′ and u′′′ are associated with Ce^4+^. The Ce^3+^/Ce^4+^ ratio for the untreated thin film surface was found to be in good agreement with prior reports on ALD grown films.^[52]^

**Table 4:** Thin film composition and oxygen to cerium ratio based on XPS analysis for an as introduced and sputtered surface of a 42 nm CeO_2_ film grown on Si(100).

	Concentration in at. % and O/Ce ratio				
	Ce	O	N	C	O/Ce
as introduced	23.3	46.2	–	30.5	1.99
sputtered	37.2	56.8	–	6.0	1.53

In light of this, analysis of the O 1s core level (see Figure 6 f) allowed to confirm the off stoichiometry of the thin film surface. Next to the O^2−^ species associated to CeO_2_ lattice oxygen at 530.0 eV[^49^, ^53^] and adsorbed OH^−^ species at 532.9 eV,^[37]^ a minor contribution from O^2−^ species arising from Ce_2_O_3_[^49^, ^53^] at 531.6 eV was found. The binding energies for all components were well within the range of positions prior reported for the respective species. In terms of the overall surface composition, determined for the as introduced surface and after 60 s of Ar^+^ sputtering, the complete absence of nitrogen impurities was noteworthy. While the carbon concentration diminished from 30.5 at.% (contribution from adventitious carbon) to roughly 6.0 at.%, the Ce/O ratio decreased from 1.99 to 1.53−a consequence of Ar^+^ ion induced reduction.^[50b]^ The results are summarized in Table S7.

**Figure 7:**
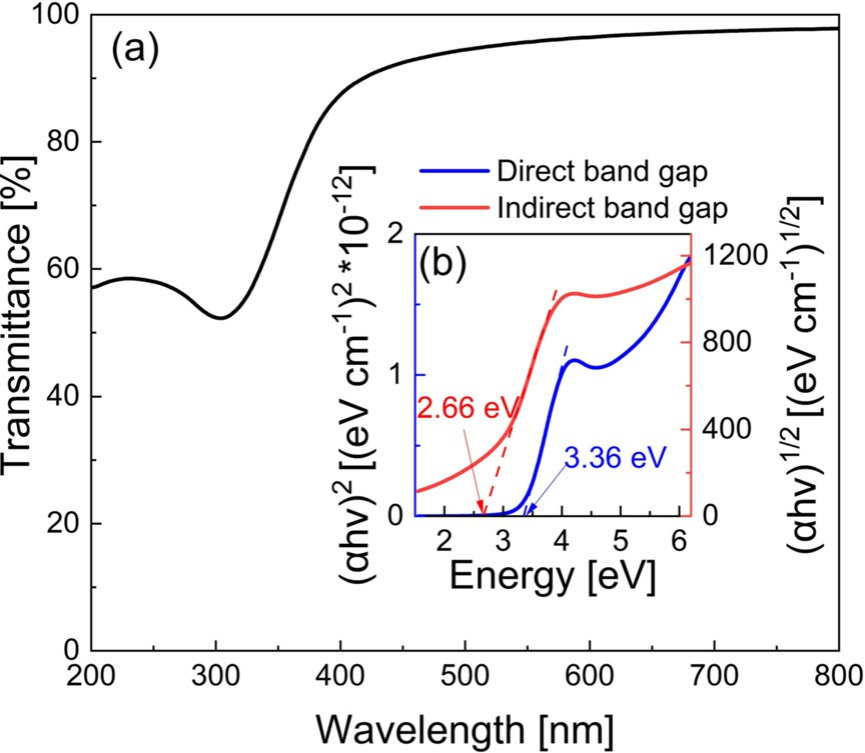
(a) Transmittance [%] of the 26 nm CeO_2_ film deposited at 160 °C on quartz. (b) Tauc plot of 26 nm CeO_2_ film deposited at 160 °C on quartz.

The bandgap of a 26 nm thick CeO_2_ film deposited on quartz was estimated by the measured UV/Visible absorption spectrum in the range 200–800 nm. A strong absorption peak is observed in the UV region at 304 nm Figure 7 a, due to the charge‐transfer transition from O(2p) to Ce(4f) orbitals in CeO_2_.^[54]^ The film shows high transparency as indicated by transmittance values of >92.5 % in the range between 450 nm −800 nm. The Tauc plot method was utilized for the direct and indirect optical bandgap calculations (see Figure 7 b). The obtained direct allowed bandgap was estimated to be 3.36 eV, and the indirect allowed bandgap was 2.66 eV, which are consistent with those reported in the literature.[^11^, ^52^]

The preliminary data for the growth of CeO_2_ thin films at mild substrate temperatures via ALD and the film characteristics shows that the initial results using [Ce(dpdmg)_3_] **1** precursor and water as a co‐reactant are highly encouraging for ALD applications. The next step is to vary the ALD process parameters to develop a reliable water assisted ALD process and then investigate the functional properties of the CeO_2_ film. Particularly the facile conversion of cerium between Ce^+3^ and Ce^+4^ oxidation states and the tunable oxygen vacancies makes it interesting for solid oxide fuel cells^[9]^ and catalytic activity for water splitting applications.^[8]^ Similar studies will be performed with other analogous precursors of Ce and Yb to compare their efficiency for new ALD process development.

## Conclusion

In the pursuit to identify new and improved precursors for ALD of Ce and Yb containing thin films, a systematic approach was undertaken tuning the ligand moieties surrounding the metals namely cerium and ytterbium. As a result, a series of cerium and ytterbium complexes [Ce(dpdmg)_3_] **1**, [Ce(bedmg)_3_] **2**, [Ce(dpdeg)_3_] **3**, [Ce(dpamd)_3_] **4**, [Ce(beamd)_3_] **5**, [Yb(dpdmg)_3_] **6**, [Yb(dpamd)_3_] **7** were successfully synthesized in good yields. The complexes can be quantitatively sublimed and exist as monomers in the gas phase. Noteworthy was the influence of the asymmetry in the molecule that could alter the melting points and thermal stabilities of the different compounds investigated. DFT study was used to analyze in detail the atomistic structure and the reactivity of **1**, **4**, **6**, and **7** in vacuum and in the presence of O_2_ and H_2_O molecules. Interestingly, in the presence of a H_2_O molecule, the bond dissociation energy is lower for **1** and **6** than for **4** and **7**, suggesting that guanidinate compounds exhibit a higher reactivity towards water, a well‐established ALD co‐reagent compared to the structurally related amidinates. The presence of O_2_ molecules had almost no effect on Yb complexes, on the contrary **1** was found to have higher reactivity toward elemental O_2_ than **4** suggesting that it could also be used as a potential precursor for ALD with molecular oxygen. Based on the promising thermal properties in terms of volatility and thermal stability as well as data inferred from the reactivity of the molecules towards water from DFT studies, these complexes certainly bear the potential to serve as new ALD precursors. Thus, proof of principle studies for water‐assisted ALD was performed with [Ce(dpdmg)_3_] **1**, yielding polycrystalline CeO_2_ thin films on Si(100) substrates. The co‐existence of Ce^3+^ and Ce^4+^ oxidation states in the films was evidenced from XPS analysis. UV/Vis analysis showed the direct allowed and indirect allowed bandgaps and hence these films could find scope for potential optical and catalytic applications which will be the focus once the ALD process is optimized. Additionally, thin CeO_2_ layers can be investigated as dielectric layers for high‐k applications. As an outlook, a comparative ALD investigation of the guanidinates vs. the amidinates for both Ce and Yb will be the focus of our future work. This study, which comprises of a rational approach undertaken towards new precursor development for Ce and Yb, enlightens the power of synthetic organometallic chemistry involving rare earths. It has always been a challenge to develop monomeric, volatile and reactive precursors for rare‐earths and hence the output of this study has substantially contributed to the expansion of the library of rare earth based precursors which to date has been particularly limited for Ce and Yb.

## Experimental Section

### Precursor synthesis

The handling and syntheses of all air and moisture‐sensitive compounds were carried out under argon atmosphere using standard Schlenk techniques. The solvents used were dried by a solvent purification system (MBraun SPS).

**Synthesis of tris(*N***,***N'*** **‐diisopropyl‐2‐dimethylamido‐guanidinato) cerium(III) [Ce(dpdmg)_3_] 1**: The synthesis procedure was adopted based on the literature.^[39b]^. *N*,*N*′‐diisopropylcarbodiimide (1.82 mL,11.76 mmol) was added dropwise to a cooled solution of lithium dimethylamine (0.6 g, 11.76 mmol) in tetrahydrofuran (THF) (25 mL). The resulting solution was stirred for to form [Li(N*i*Pr)_2_CNMe_2_]. In another flask THF was added to anhydrous CeCl_3_ (0.946 g, 3.91 mmol) and stirred to form a suspension. [Li(N*i*Pr)_2_CNMe_2_] was added to the suspension and the mixture was refluxed at 60 °C. The resulting solution was cooled to room temperature (RT) and the solvent was removed, and the product was extracted in hexane while the precipitated LiCl was filtered off. After removal of the solvent under reduced pressure, the resulting yellow product was sublimed in vacuum at 120 °C that yielded 2.09 g of a yellow crystalline product. Crystals suitable for single‐crystal X‐ray analysis were obtained by sublimation. Yield: 82.07 %. ^1^H NMR (400 MHz, [D_6_]C_6_D_6_, RT, paramagnetic) *δ*=9.44 [6 H, (NC*H*(CH_3_)_2_)_2_CNMe_2_], 5.78 [18 H, (N*i*Pr)_2_CN(C*H*_3_)_2_], −10 to 4 [broad, (NCH(C*H*_3_)_2_)_2_CNMe_2_]. ^13^C NMR (400 MHz, [D_6_]C_6_D_6_, RT) *δ*=162.37 [(N*i*Pr)_2_ *C*NMe_2_], 46.03 [(N*C*H(CH_3_)_2_)_2_CNMe_2_], 44.48 [(N*i*Pr)_2_CN(*C*H_3_)_2_], 22.23 ppm [(NCH(*C*H_3_)_2_)_2_CNMe_2_]. EI‐MS (70 eV) [ML_3_^+^=CeL_3_^+^, L={(*i*PrN)_2_CNMe_2_}] *m*/*z* (rel. int.%)=650(14.7) [CeL_3_^+^], 524.2(5.2) [CeL_3_‐2*i*Pr‐NMe_2_^+^], 480(35.3) [CeL_2_^+^], 398.2(17.1) [CeL_2_‐2*i*Pr^+^], 354.1(71.1) [CeL_2_‐2*i*Pr‐NMe_2_^+^], 311.4(13.2) [CeL^+^], 267.0 (6.01) [CeL‐*i*Pr^+^], 171(5.84) [L^+^], 69(100) [*i*Pr‐NC^+^], 44(38.8) [Me_2_N^+^ or C_3_H_8_^+^], 43(75.5) [*i*Pr^+^]. FT‐IR (cm^−1^) $\tilde{\nu }$ =2955(m), 2862(m), 1496(m), 1451(s), 1377(s), 1313(s), 1236(w), 1169(s), 1121(m), 1038(s), 733(w), 693(w), 534(m), 441(m). Elemental analysis calcd (%) for C_27_H_60_N_9_Ce: C, 49.82; H, 9.29; N, 19.37. Found (%): C, 49.09; H, 9.34; N, 19.00.

**Synthesis of tris (*N***,***N'*** **‐butylethyl‐2‐dimethylamido‐guanidinato) cerium(III) [Ce(bedmg)_3_] 2**: Following the same procedure as for **1**, [Li(N*t*Bu)(NEt)CNMe_2_] was prepared by *N*‐*tert‐*butyl‐*N*′‐ethylcarbodiimide (1 mL, 6.45 mmol) and LiNMe_2_ (0.329 g, 6.45 mmol) in THF (15 mL). [Li(N*t*Bu)(NEt)CNMe_2_] was added to the CeCl_3_ (0.528 g, 2.14 mmol) suspension in THF (15 mL) and refluxed at 60 °C. Following the same work up as described above, 1 g of yellow powder was obtained. Yield 71.64 %. ^1^H NMR (400 MHz, [D_6_]C_6_D_6_, RT, paramagnetic) *δ*=7.52 [24 H, (N*t*Bu)(NC*H_2_*CH_3_)CN(C*H*_3_)_2_], −2.83 [27 H, (NC(C*H_3_*)_2_)(NEt)CNMe_2_], −5.72 ppm [9 H, (N*t*Bu)(NCH_2_C*H_3_*)CNMe_2_]. ^13^C NMR (400 MHz, [D_6_]C_6_D_6_, RT) *δ*=176.32 [(N*t*Bu)(NEt)*C*NMe_2_], 53.20 [(N*C*(CH_3_)_2_)(NEt)CNMe_2_], 47.41 [((N*t*Bu)(NEt)CN(*C*H_3_)_2_], 41.05 [(N*t*Bu)(N*C*H_2_CH_3_)CNMe_2_ ], 28.88 [(NC(*C*H_3_)_2_)(NEt)CNMe_2_], 10.87 ppm [(N*t*Bu)(NCH_2_ *C*H_*3*_)CNMe_2_]. EI‐MS (70 eV) [ML_3_^+^=CeL_3_^+^, L={(N*t*Bu)(NEt)CNMe_2_}] *m*/*z* (rel. int.%)=650(9.41) [CeL_3_^+^], 524.2(10.96) [CeL_3_ ‐*t*Bu ‐Et ‐NMe_2_^+^], 480(13.66) [CeL_2_^+^], 398.2(71.60) [CeL_2_‐*t*Bu ‐Et ^+^], 354.1(58.81) [CeL_2_‐*t*Bu ‐Et ‐NMe_2_^+^], 311.4(15.91) [CeL^+^], 253.0 (9.03) [CeL‐*t*Bu^+^], 171.2(1.83) [L], 83.0(60.25) [*t*Bu‐NC^+^], 57.0(100) [Et‐NC^+^], 44.0(19.88) [Me_2_N^+^]. FT‐IR (cm^−1^) $\tilde{\nu }$ =2954(m), 2861(m), 1477(s), 1437(s), 1383(s), 1337(s), 1200(s), 1142(m), 1109(s), 1068(s), 997(m), 785(w), 711(m), 545(m). Elemental analysis calcd (%) for C_27_H_60_N_9_Ce: C, 49.82; H, 9.29; N, 19.37. Found (%): C, 49.32; H, 9.33; N, 19.46.

**Synthesis of tris (*N***,***N'*** **‐diisopropyl‐2‐diethylamido‐guanidinato) cerium(III) [Ce(dpdeg)_3_] 3**: Following the same procedure as for **1**, [Li(N*i*Pr)_2_CNEt_2_]. was prepared by *N*,*N*′‐diisopropylcarbodiimide (1.5 mL, 9.69 mmol) and LiNEt_2_ (0.765 g, 9.69 mmol) in THF. (40 mL) [Li(N*i*Pr)_2_CNEt_2_] was added to the CeCl_3_ (0.796 g, 3.23 mmol) suspension in THF (40 mL) and refluxed at 60 °C. Following the same work up as described above, 2.09 g of yellow product was obtained. Yield 87.98 %. ^1^H NMR (400 MHz, [D_6_]C_6_D_6_, RT, paramagnetic) *δ*=9.97 [6 H, (NC*H*(CH_3_)_2_)_2_CNEt_2_], 6.33 and 5.70 [12 H, (N*i*Pr)_2_CN(C*H_2_*CH_3_)_2_], 3.12 [18 H, (N*i*Pr)_2_CN(CH_2_C*H_3_*)_2_], 2.53 and −6.47 ppm [18 H and 18 H, (NCH(C*H*_3_)_2_)_2_CNEt_2_]. ^13^C NMR (400 MHz, [D_6_]C_6_D_6_, RT) *δ*=160.87 [(N*i*Pr)_2_ *C*NEt_2_], 47.27 [(N*i*Pr)_2_CN(*C*H_2_CH_3_)_2_], 46.21 [(N*C*H(CH_3_)_2_)_2_CNEt_2_], 26.55 and 17.83 [(NCH(*C*H_3_)_2_)_2_CNEt_2_], 16.96 ppm [(N*i*Pr)_2_CN(CH_2_ *C*H_3_)_2_]. EI‐MS (70 eV) [ML_3_^+^=CeL_3_^+^, L={(*i*PrN)_2_CNEt_2_}] *m*/*z* (rel. int.%)=734(46.8) [CeL_3_^+^], 536.3(40.8) [CeL_2_^+^], 464(8.46) [CeL_2_‐NEt_2_^+^], 410(87.9) [CeL +NEt_2_^+^], 337.1(49.4) [CeL^+^], 282.0 (48.42) [CeL‐CH_2_CH_2_^+^], 199(58) [L^+^], 69(100) [*i*Pr‐NC^+^], 43(100) [*i*Pr^+^]. FTIR (cm^−1^) $\tilde{\nu }$ =2958(s), 2862(m), 1463(s), 1437(s), 1410(s), 1374(s), 1305(s), 1214(s), 1169(s), 1129(m), 1054(s), 925(w), 708(w), 546(w), 443(m). Elemental analysis calcd (%) for C_33_H_72_N_9_Ce: C, 53.92; H, 9.87; N, 17.15. Found (%): C, 53.95; H, 10.65; N, 16.66.

**Synthesis of tris(*N***,***N'*** **‐diisopropyl‐acetamidinato) cerium(III) [Ce(dpamd)_3_] 4**: Following the same procedure as for **1**, [Li(N*i*Pr)_2_CMe] was prepared by *N*,*N*′‐diisopropylcarbodiimide (2.5 mL, 16.14 mmol) and 1.6 m LiMe in hexane (10 mL, 16.14 mmol) in THF (50 mL). [Li(N*i*Pr)_2_CMe] was added to the CeCl_3_ (1.33 g, 5.38 mmol) suspension in THF (40 mL) and refluxed at 60 °C. Following the same work up as described above, 2.80 g of yellow product was obtained. Yield 92.3 %. ^1^H NMR (300 MHz, [D_6_]C_6_D_6_, RT, paramagnetic) *δ*=11.01 [6 H, (NC*H*(CH_3_)_2_)_2_CMe], 10.81 [9 H, (N*i*Pr)_2_C(C*H*_3_)], −3.99 [ 36 H, (NCH(C*H*_3_)_2_)_2_CMe]. ^13^C NMR (300 MHz, [D_6_]C_6_D_6_, RT) *δ*=189.31 [(N*i*Pr)_2_ *C*Me], 52.58 [(N*C*H(CH_3_)_2_)_2_CMe], 25.83 [(N*i*Pr)_2_C(*C*H_3_)], 22.20 ppm [(NCH(*C*H_3_)_2_)_2_CMe]. EI‐MS (70 eV) [ML_3_^+^=CeL_3_^+^, L={(*i*PrN)_2_CMe}] *m*/*z* (rel. int.%)=563(38.84) [CeL_3_^+^], 422.2(100) [CeL_2_^+^], 296.1(20.5) [CeL+NH^+^], 280(3.4) [CeL^+^], 197(10.9) [CeN*i*Pr^+^], 142(17.5) [L^+^].FT‐IR (cm^−1^) $\tilde{\nu }$ =2958(s), 2925(m), 2864(m), 1480(s), 1141(s), 1374(s), 1328(s), 1309(s), 1193(s), 1169(s), 1120(s), 1046(m), 1013(m), 797(s), 637(s), 566(s), 537(s). Elemental analysis calcd (%) for C_24_H_51_N_6_Ce: C, 51.13; H, 9.12; N, 14.91. Found (%): C, 51.00; H, 8.79; N, 14.35.

**Synthesis of tris (*N***,***N'*** **‐butylethyl‐acetamidinato) cerium(III) [Ce(beamd)_3_] 5**: Following the same procedure as for **1**, [Li(N*t*Bu)(NEt)CMe] was prepared by *N*‐*tert*‐butyl‐*N*′‐ethylcarbodiimide (0.5 mL, 3.22 mmol) and 1.6 m LiMe in hexane (2.02 mL, 3.22 mmol) in THF (20 mL). [Li(N*t*Bu)(NEt)CMe] was added to the CeCl_3_ (0.264 g, 1.07 mmol) suspension in THF (15 mL) and refluxed at 60 °C. Following the same work up as described above, 0.495 g of yellow powder was obtained. Yield 81.68 %. ^1^H NMR (400 MHz, [D_6_]C_6_D_6_, RT, paramagnetic) *δ*=11.02 [9 H, (N*t*Bu)(NEt)C(C*H*_3_)], 6.36 [6 H, (N*t*Bu)(NC*H_2_*CH_3_)CMe], −3.27 [27 H, (NC(C*H_3_*)_2_)(NEt)CMe], −6.16 ppm [9 H, (N*t*Bu)(NCH_2_C*H_3_*)CMe]. ^13^C NMR (400 MHz, [D_6_]C_6_D_6_, RT) *δ*=189.19 [(N*t*Bu)(NEt)*C*Me], 54.85 [(N*C*(CH_3_)_2_)(NEt)CMe], 44.50 [(N*t*Bu)(N*C*H_2_CH_3_)CMe], 31.40 [((N*t*Bu)(NEt)C(*C*H_3_)], 29.06 [(NC(*C*H_3_)_2_)(NEt)CMe], 10.83 ppm [(N*t*Bu)(NCH2*C*H_*3*_)CMe]. EI‐MS (70 eV) [ML_3_^+^=CeL_3_^+^, L={(N*t*Bu)(NEt)CMe}] *m*/*z* (rel. int.%)=563.4(44.6) [CeL_3_^+^], 422.2(100) [CeL_2_^+^], 296.1(6.83) [CeL+NH^+^], 280(5.9) [CeL^+^], 142.1(18.8) [L^+^]. FT‐IR (cm^−1^) $\tilde{\nu }$ =2958(m), 2862(m), 1483(s), 1408(s), 1335(s), 1207(s), 1150(s), 1084(m), 1028(m), 979(m), 810(m), 758(m), 634(m), 568(w), 472(w). Elemental analysis calcd (%) for C_24_H_51_N_6_Ce: C, 51.13; H, 9.12; N, 14.91. Found (%): C, 50.16; H, 8.91; N, 15.18.

**Synthesis of tris(*N***,***N'*** **‐diisopropyl‐2‐dimethylamido‐guanidinato) Ytterbium(III) [Yb(dpdmg)_3_] 6**: The synthesis procedure was done based on the isostructural [Er(dpdmg)_3_].^[40b]^ *N*,*N*′‐diisopropylcarbodiimide (6.02 mL, 38.88 mmol) was added a suspension of lithium dimethylamine (2.09 g, 38.88 mmol) in diethyl ether (85 mL). The resulting solution was stirred to form [Li(N*i*Pr)_2_CNMe_2_]. [Li(N*i*Pr)_2_CNMe_2_] was added to a cooled solution of YbCl_3_ (3.97 g, 13.56 mmol) in THF (30 mL) and stirred. The solvent was removed under reduced pressure and was extracted in hexane and precipitated LiCl was filtered off. A saturated solution in hexane was prepared to obtain pale yellow crystals at −30 °C suitable for X‐ray analysis. Yield: 70.92 %. EI‐MS (70 eV) [ML_3_^+^=YbL_3_^+^, L={(*i*PrN)_2_CNMe_2_}] *m*/*z* (rel. int.%)=684.8(1.95) [YbL_3_^+^], 513.4(92.46) [YbL_2_^+^], 468(66.53) [YbL_2_‐NMe_2_^+^], 343.4(94.21) [YbL^+^], 301.1 (36.18) [YbL‐*i*Pr^+^], 170.2(24.99) [L^+^], 69(100) [*i*Pr‐NC^+^], 43(94.33) [*i*Pr^+^]. FT‐IR (cm^−1^) $\tilde{\nu }$ =2960(m), 2917(m), 2865(m), 1464(s), 1449(s), 1389(s), 1315(s), 1215(m), 1185(m), 1134(m), 1044(s), 929(w), 833(w), 734(m), 692(m), 539(m), 445(m). Elemental analysis calcd (%) for C_27_H_60_N_9_Yb: C, 47.42; H, 8.84; N, 18.43. Found (%): C, 47.12; H, 9.51; N, 18.43.

**Synthesis of tris(*N***,***N'*** **‐diisopropyl‐acetamidinato) Ytterbium (III) [Yb(dpamd)_3_] 7**: Following the same procedure as for **6**, [Li(N*i*Pr)_2_CMe] was prepared by *N*,*N*′‐diisopropylcarbodiimide (2.5 mL, 16.14 mmol) and 1.6 m LiMe in hexane (10.09 mL, 16.14 mmol) in diethyl ether (70 mL). [Li(N*i*Pr)_2_CMe] was added to a cooled solution of YbCl_3_ (1.5 g, 5.38 mmol) in THF (100 mL) and stirred. Following the same work up as described above, a light green product was obtained which was further purified by sublimation to obtain 2.8 g of **7**. Yield 87.27 %. EI‐MS (70 eV) [ML_3_^+^=YbL_3_^+^, L={(*i*PrN)_2_CMe}] m/*z* (rel. int.%)=597.5(7.03) [YbL_3_^+^], 455.3(100) [YbL_2_^+^], 440.2(8.61) [YbL_2_‐Me^+^], 313(28.90) [YbL^+^], 142(28.6) [L^+^].FT‐IR (cm^−1^) $\tilde{\nu }$ =2959(s), 2928(m), 2864(m), 1465(s), 1415(s), 1374(s), 1331(s), 1312(s), 1206(s), 1171(s), 1137(m), 1121(s), 1052(m), 1015(m), 926(w), 802(m), 618(m), 570(m), 546(m), 423(m). Elemental analysis calcd (%) for C_24_H_51_N_6_Yb: C, 48.3; H, 8.61; N, 14.08. Found (%): C, 46.51; H, 8.99; N, 13.78.

### Precursor characterization

^1^H, ^13^C nuclear magnetic resonance (NMR) and heteronuclear single quantum coherence (HSQC) spectra were measured on a Bruker AV III 400 spectrometer and a Bruker AV III 300 spectrometer at 298 K. All signals were referenced to the residual proton signals of deuterated solvents and corrected to the TMS (tetramethylsilane) standard values. Temperature dependent NMR was recorded on a Bruker Advance DPX 250 spectrometer. For that, the compound was filled in a heavy‐walled NMR tube and dissolved in a freshly prepared degassed [D_8_]toluene and sealed by melting. The NMR spectra received were further analyzed with the MestReNova software.^[55]^ CHNS elemental analysis (EA) was performed using a vario Micro cube from Elementar Analysensysteme. Electron impact ionization mass spectrometry (EI‐MS) was performed using a VG Instruments Autospec instrument at an ionization energy of 70 eV. Fourier‐transform infrared (FTIR) spectroscopy was performed between 400–4000 cm^−1^ using a Spectrum Two instrument from PerkinElmer with an attenuated total reflectance (ATR) unit in an argon‐filled glove box. Thermogravimetric analysis (TGA) was carried out by using a Netzsch STA 409 PC/PG at ambient pressure (sample size ≈15 mg in a round alumina crucible having diameter 6.15 mm. For TGA, a heating ramp of 5 °C min^−1^ and nitrogen (AirLiquide, 99.998 %) flow rate of 90 sccm was used. Melting points were determined by simultaneous differential scanning calorimetry (DSC) (mW mg^−1^).

Crystals of suitable quality were selected from perfluoropolyether oil on a microscope slide under an optical microscope with a polarized light source and was immediately mounted in a liquid nitrogen cooled gas stream of a diffractometer. A crystal of [Ce(dpdmg)_3_] **1** was measured on an Agilent Technologies SuperNova diffractometer with an Atlas CCD detector and Cu Kα radiation from a microfocus X‐ray source with multilayer X‐ray optics and [Yb(dpdmg)_3_] **6** on an Oxford Diffraction Xcalibur2 diffractometer with a Sapphire2 CCD and Mo Kα radiation. The diffraction data were processed with CrysAlisPro.^[56]^ Empirical absorption correction was done using spherical harmonics, implemented in SCALE3 ABSPACK scaling algorithm. The crystal structures were solved and refined by using SHELXL,^[57]^ SHELXLe‐2014^[58]^ and OLEX2.^[59]^ Deposition Numbers 2023020 ([Ce(dpdmg)_3_] **1**) and 2023021 ([Yb(dpdmg)_3_] **6**) contain(s) the supplementary crystallographic data for this paper. These data are provided free of charge by the joint Cambridge Crystallographic Data Centre and Fachinformationszentrum Karlsruhe Access Structures service www.ccdc.cam.ac.uk/structures. 

### Computational method

The ground state electronic wave function of each molecule was calculated self‐consistently within Kohn–Sham Density Functional Theory (DFT) using the TURBOMOLE suite of quantum chemical programs.^[60]^ These calculations were performed by using the hybrid PBE0 functional, which incorporates 25 % exact HF exchange,^[61]^ and a polarized split valance basis set, denoted def‐SV(P).^[62]^ An effective core potential is used for the Ce and Yb metal sites with 28 core electrons on both rare earths. A fine integration grid (m3) was used and the SCF convergence criterion was set to 10^−6^ Ha. Precursor atomic structures are prepared from the experimental cif files in Materials Studio 8.0 and exported in *xyz* format; all structures are freely available in a GitHub repository.^[63]^ Convergence criteria for the geometry was set to 10^−3^ Ha.

The energy needed to lose the first ligand is calculated using [Eq. 1]:

(1) $$E{}^{\mathrm{Ligand}}=\left(E{}_{L}+E{}_{P-1L}\right)-E{}_{P}$$

*E_L_*: Computed total energy of one free ligand; *E_P_*: Computed total energy of the precursor molecule; *E*_*P−1L*_: Computed total energy of the precursor without one ligand

For the example of [Ce(dpdmg)_3_] **1** [Eq. 2]:

(2) $$E{}^{\mathrm{Ligand}}=\left(E{}_{\mathrm{dpdmg}}+E{}_{\mathrm{Ce}\left(\mathrm{dpdmg}\right){}_{2}}\right)-E{}_{\mathrm{Ce}\left(\mathrm{dpdmg}\right){}_{3}}$$

 

### Thin‐film deposition

Cerium oxide thin films were deposited using tris(*N*,*N*′‐diisopropyl‐2‐dimethylamido‐guanidinato) cerium(III) [Ce(dpdmg)_3_] **1** as the precursor and deionized water as the co‐reactant. The synthesis of [Ce(dpdmg)_3_] **1** was upscaled to large batches of ca. 10 g for preliminary ALD experiments. All the depositions were carried out in a F‐120 ASM Microchemistry flow‐type ALD reactor on 2 cm * 2 cm silicon and quartz substrates. The reactor is setup into eight zones to achieve a gradually increasing temperature profile from precursor zone to deposition zone. Nitrogen (99.999 % purity) gas was implemented as a carrier and purging gas at 300 sccm. The sublimation temperature for [Ce(dpdmg)_3_] **1** was set 140 °C (zone 2) and H_2_O was maintained at room temperature. The pulse purge sequence applied for thickness dependent studies (illustrated in the SI in Figure S14) at deposition temperature of 160 °C (zone 7) is precursor **1** pulse (8 s)/N_2_ purge (30 s)/H_2_O pulse (3 s)/N_2_ purge (30 s).

### Thin‐film characterization

Film thickness was measured by X‐ray reflectivity (XRR), and the film crystallinity was assessed by grazing incidence X‐ray diffraction (GIXRD) using a Panalytical XPert diffractometer and Cu Kα source on silicon substrates. The GIXRD fitting was performed by using Reflex module in the Materials Studio 8.0 (BIOVIA Software Inc., USA). The background for GIXRD was calculated with a Gaussian width of 0.01 and polynomial order of 2 and subtracted. The reference GIXRD pattern was computed using cubic CeO_2_ (ICDD: 04–016–4620). The Rietveld refinement method with a small degree of Zero shift and peak boarding was employed to achieve the fitting of GIXRD pattern;^[64]^ a BraggBrentano function^[65]^ was used for instrument geometry, Thompson‐Cox‐Hastings^[66]^ for peak profile, and Finger‐Cox‐Jephcoat function^[67]^ for Asymmetry correction. The X′Pert Reflectivity program v1.3 from PANalytical was utilized for the fitting of the XRR patterns. X‐ray photoelectron spectroscopy (XPS) was carried out in a PHI 5000 instrument. The X‐ray source was operated at 10 kV and 24.6 W using Al Kα (1486.6 eV) radiation with a 45° electron takeoff angle. The kinetic energy of electrons was analyzed with a spherical Leybold EA−10/100 analyzer using a pass energy of 18 eV. The samples were analyzed by core level scans for peaks of interest. The step width was adjusted to 0.05 eV for the core level scans. Spectra were recorded prior to and after sputter cleaning (1 min. 2 kV 2×2). All binding energies of cerium Ce 3d and oxygen O 1s were referenced to adventitious carbon C 1s at 284.8 eV. The analysis chamber pressure was maintained at <10^−8^ mbar. The deconvolution analysis was completed with a Shirley background processing and Gaussian functions using UniFit 2017 software. The UV/Vis absorption spectra in absorbance mode was collected by the application of Shimadzu UV/2600 spectrometer using wavelength in the range of 200–800 nm on the quartz substrate.

## Supporting information

As a service to our authors and readers, this journal provides supporting information supplied by the authors. Such materials are peer reviewed and may be re‐organized for online delivery, but are not copy‐edited or typeset. Technical support issues arising from supporting information (other than missing files) should be addressed to the authors.

Supplementary
