# Supplementary material for: Rational Development of Guanidinate and Amidinate Based Cerium and Ytterbium Complexes as Atomic Layer Deposition Precursors: Synthesis, Modeling, and Application
Source: Chemistry. 2021 Jan 20;27(15):4913–26. doi: 10.1002/chem.202003907 (PMC7986905; doi:10.1002/chem.202003907)
Supplement: Supplementary file 1 — Supplementary [file CHEM-27-4913-s001.pdf]

Chemistry–A European Journal

Supporting Information

Rational Development of Guanidinate and Amidinate Based Cerium and Ytterbium Complexes as Atomic Layer Deposition Precursors: Synthesis, Modeling, and Application

Parmish Kaur,[a] Lukas Mai,[a] Arbresha Muriqi,[b] David Zanders,[a] Ramin Ghiyasi,[c] Muhammad Safdar,[c] Nils Boysen,[a] Manuela Winter,[a] Michael Nolan,[b] Maarit Karppinen,[c] and Anjana Devi*[a]

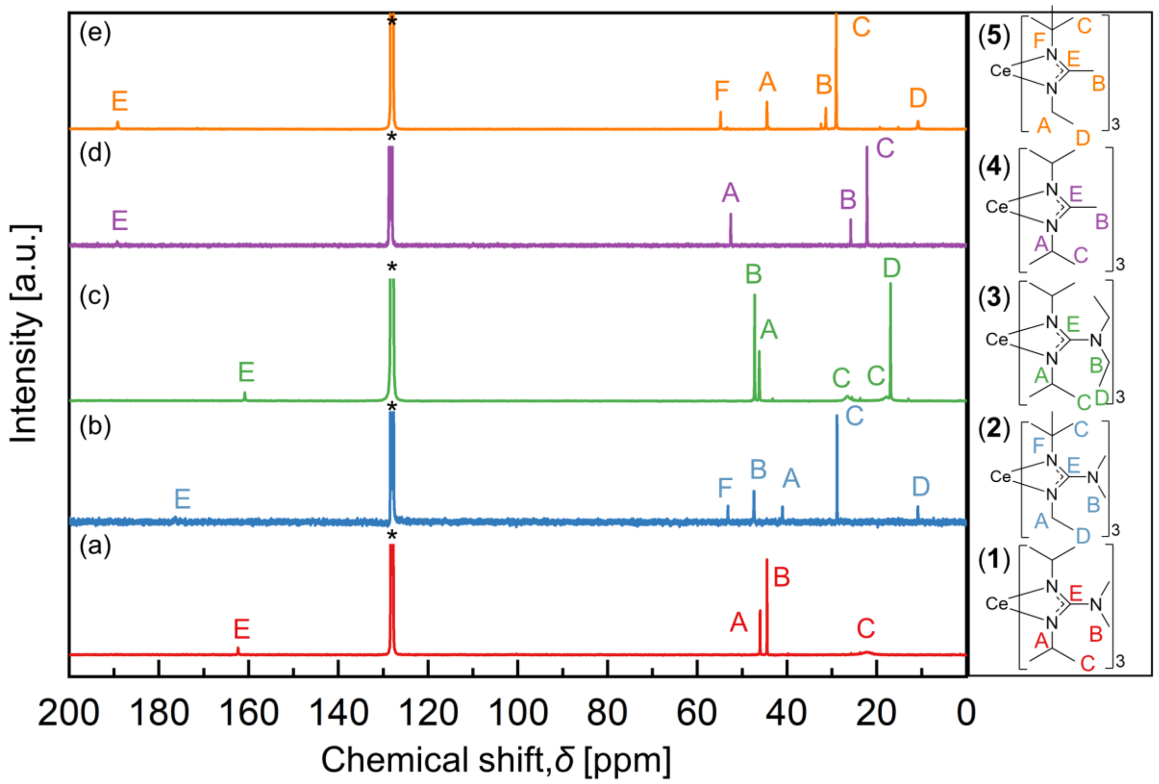

Figure S1 ^{13}C NMR of complexes **1 - 5** ((a),(b),(c),(e) C_6D_6-400 MHz-RT and (c) C_6D_6-300 MHz-RT). * C_6D_6 at 128.06 ppm

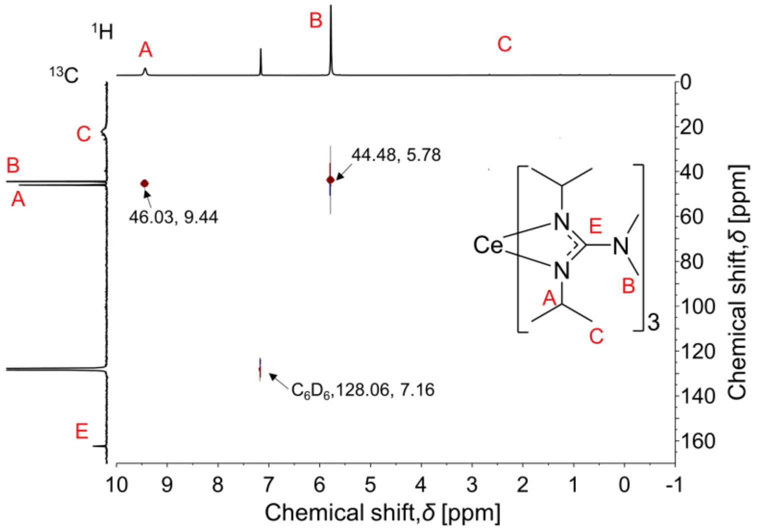

Figure S2 HSQC (^{1}H-^{13}C) spectra of [Ce(dpdmg)$_3$] **1** (C_6D_6-400 MHz-RT)

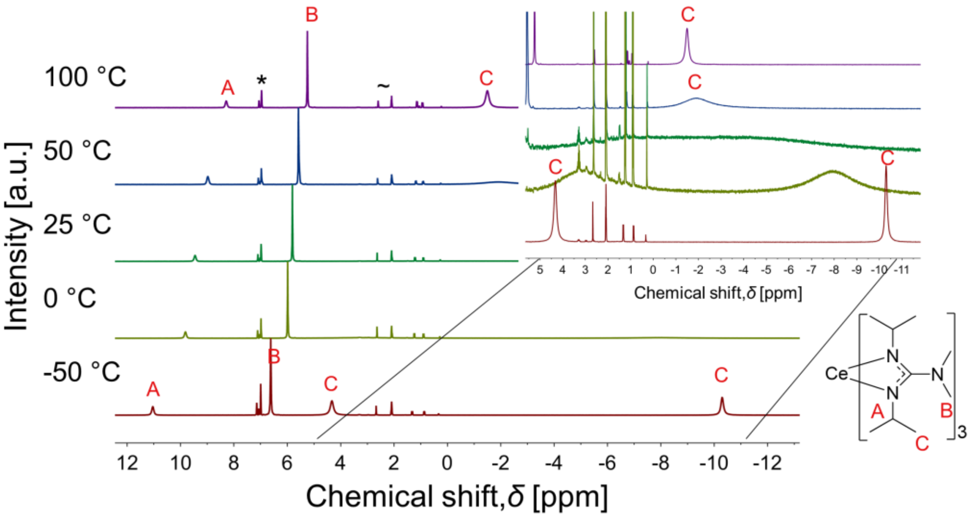

Figure S3 Temperature dependent-NMR of [Ce(dpdmg)$_3$] **1** ([D$_8$]Toluene-250 MHz) -50 °C to 100 °C (* toluene and ~ unreacted ligand)

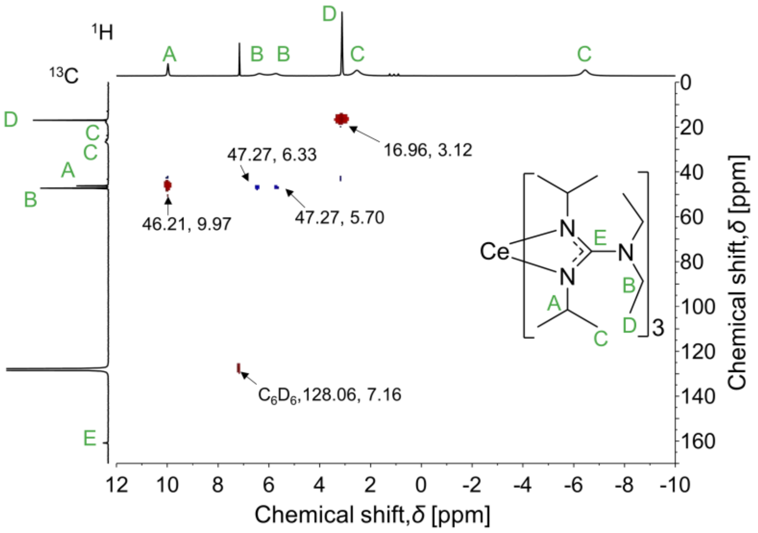

Figure S4 HSQC (^{1}H-^{13}C) spectra of [Ce(dpdeg)$_3$] **3** (C_6D_6-400 MHz-RT)

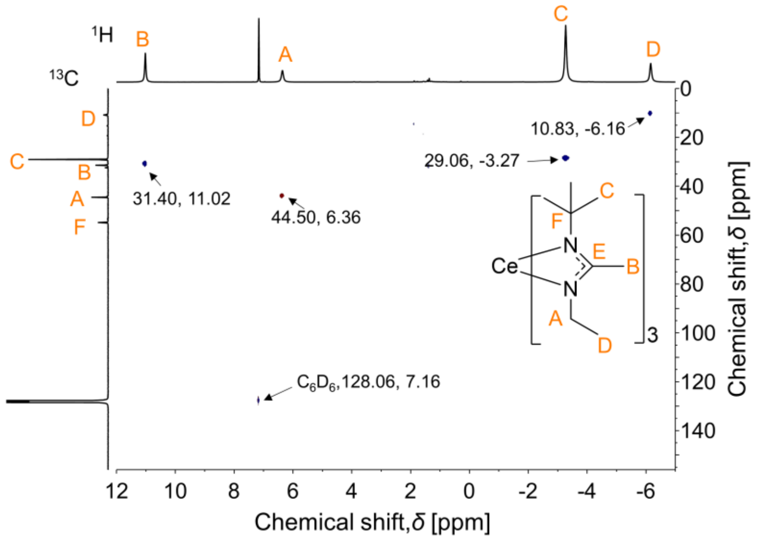

Figure S5 HSQC (^{1}H-^{13}C) spectra of [Ce(beamd)$_3$] **5** (C_6D_6-400 MHz-RT)

Detailed NMR interpretation:

Figure 1 in the manuscript shows ^{1}H NMR spectra for the complexes **1**-**5**.For complex **1** (Figure 1a), the ^{1}H NMR shows a peak with a chemical shift of 9.44 ppm (peak A) and an integral of six, which can be assigned to the CH protons of the *i*Pr group. At 5.78 ppm, peak B can be found with an integral of 18 protons, corresponding to the protons of the methyl group in the C-NMe_2 moiety. A very broad peak in the ^{1}H-NMR spectrum is detected with an integral of 36 protons in a range of -10 ppm to 4 ppm and can be assigned to the protons of the methyl groups of the *i*Pr rest. The corresponding carbon peak is found at 22.23 ppm in the ^{13}C-NMR. However, HSQC does not evidence a correlation for it, most probably because of the paramagnetic broadening of the peak. For the other peaks, the HSQC (Figure S2) confirmed that the corresponding carbons attached to the hydrogens described observed at a chemical shift of 46.37 ppm and 44.79 ppm in the ^{13}C-NMR (Figure S1a), respectively. The most deshielded carbon peak from the N-C-N backbone is found at 160.37 ppm which does not show any correlation to protons. In complex **2**, the ^{1}H peak at 7.52 ppm (peak A and B) arises from two overlaying proton signals which are assigned to the protons of the CH_2 group in the Et moiety and the protons of the methyl groups in C-NMe_2 groups (Figure 1b). The integration matches and sums up to 24 protons. The chemical shifts of the signals for the CH_3 protons of the *t*Bu groups and the CH_3 protons of the Et groups are found at -2.83 ppm (peak C) with an integral of 27 and at 5.72 ppm (peak D) with an integral of 9 respectively. All the chemical shifts for ^{13}C NMR were assignable, as depicted in Figure S1b. In complex **3** (Figure 1c), the peak at a chemical shift of 9.97 ppm (peak A) with an integral of six was observed for the CH protons of the *i*Pr groups. The chemically inequivalent CH_2 protons for the C-NEt_2 moiety are observed where the peak B is found at a chemical shift of 6.33 ppm and 5.70 ppm having an integral of twelve. The proof that the two signals arise from the same group is found in the HSQC spectrum (Figure S4), showing that the two proton signals at 6.33 ppm and 5.70 ppm (peak B) are only correlating with the signal at 47.27 ppm (peak B) in the ^{13}C NMR. A single sharp peak at 3.12 ppm (peak D) having an integral of 18 was observed for the CH_3 protons of C-NEt_2 moiety and could originate from the free rotation of the bond. The CH_3 protons of the *i*Pr group are found to have two peaks at 2.53 ppm (peak C) and -6.47 ppm (peak C) each of them having an integral of 18 which could be the result of the hindered rotation of the *i*Pr groups that are thus having a chemically inequivalent environment. Complex **4** was reported earlier and our findings were in agreement with those by Liyong Du *et al.*[1]. We have also assigned the ^{13}C peaks to the complex (Figure S1d). In a similar way, all the protons (Figure1e) and the carbon signals were clearly visible for complex **5** (Figure S1e). The signal of the CH_3 protons of the C-Me moiety and the CH_2 protons of the Et moiety are upfield shifted and found at 11.02 ppm (peak B) with an integral of nine and 6.36 ppm (peak A) with an integral of six, respectively, whereas the CH_3 protons of the *t*Bu group and the CH_3 protons of the Et group are downfield shifted at -3.27 ppm (peak C) with an integral of 27 and -6.16 ppm (peak D) with an integral of nine, respectively. HSQC (Figure S5) was used to clearly identify the bonds between proton and carbon atoms. There was no correlation peak found for the peak at 54.85 ppm and hence, it is assigned to the tertiary carbon of *t*Bu group. The peaks at chemical shifts of 44.50 ppm, 31.40 ppm, 29.06 ppm, and 10.83 ppm are assigned to the methylene group of the Et-group, the methyl group of the C-Me backbone, the methyl group of the *t*Bu group, and the methyl group of the ethyl-group, respectively.

[1] L. Du, K. Wang, Y. Zhong, B. Liu, X. Liu, Y. Ding, *J. Mater. Sci.* **2020**, *55*, 5378–5389.

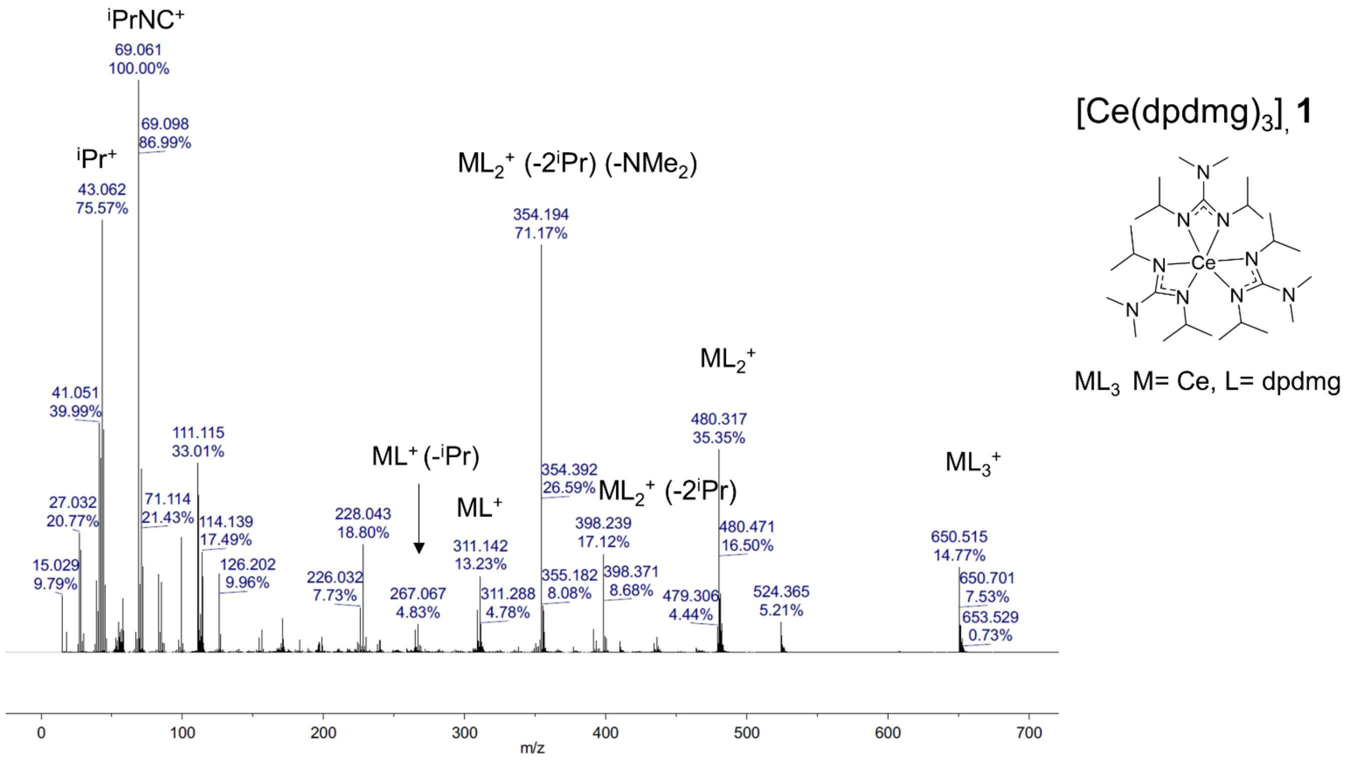

Figure S6 Mass spectrum of [Ce(dpdmg)3] **1** (EI-MS, 70 eV).

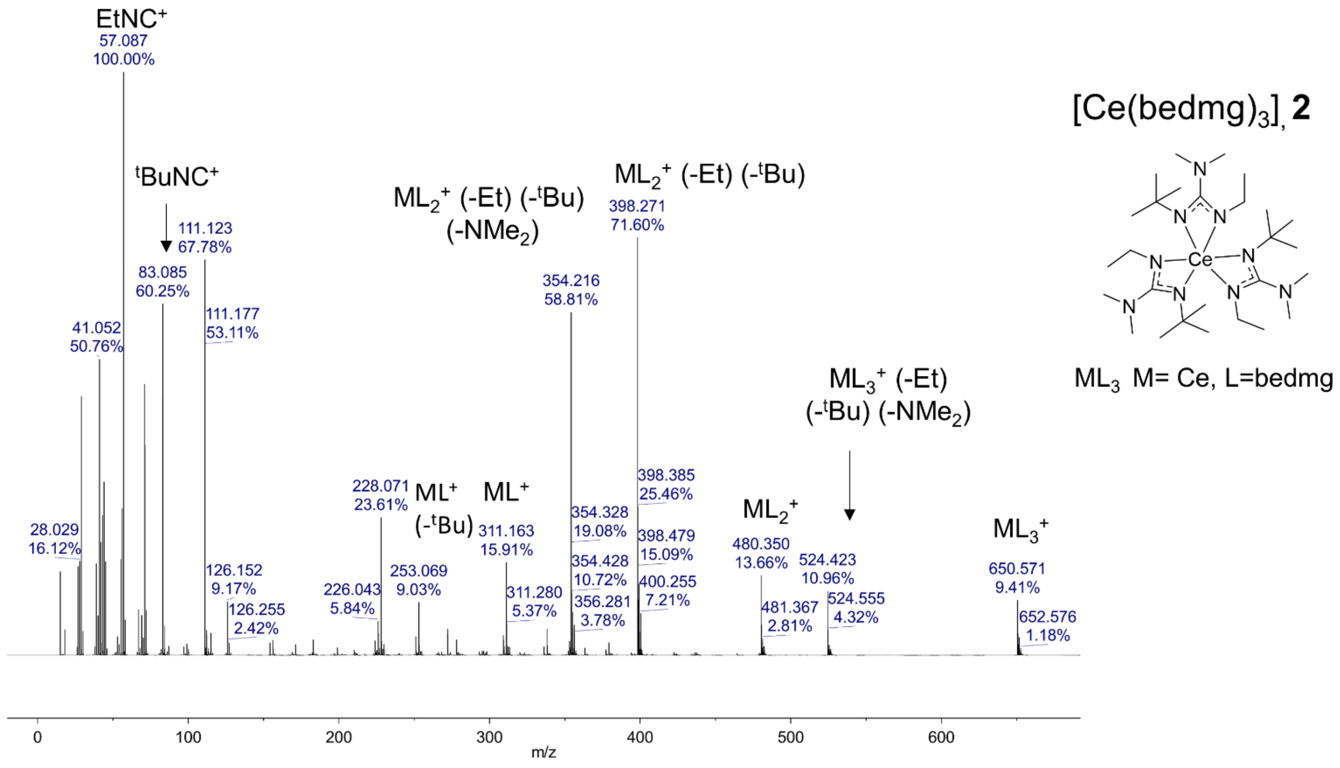

Figure S7 Mass spectrum of [Ce(bedmg)3] **2** (EI-MS, 70 eV).

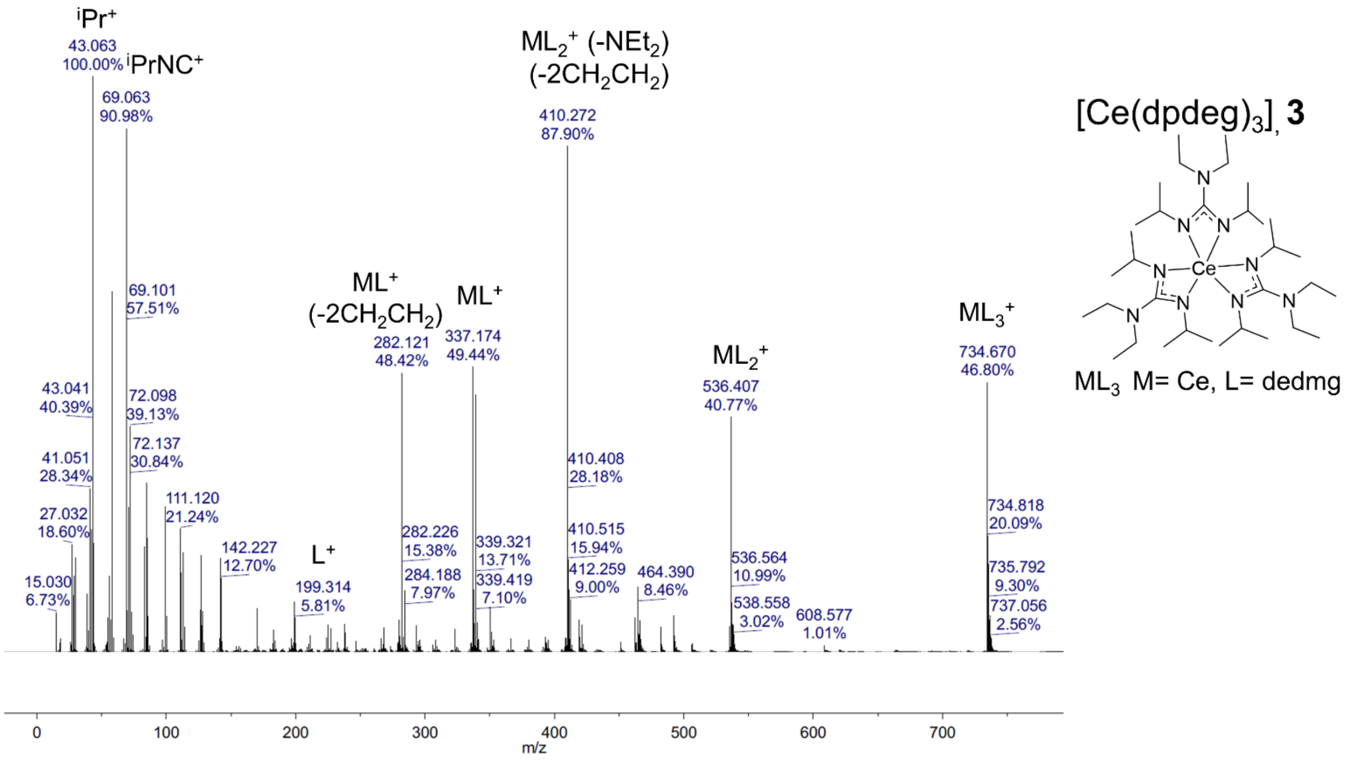

Figure S8 Mass spectrum of [Ce(dpdeg)3] **3** (EI-MS, 70 eV).

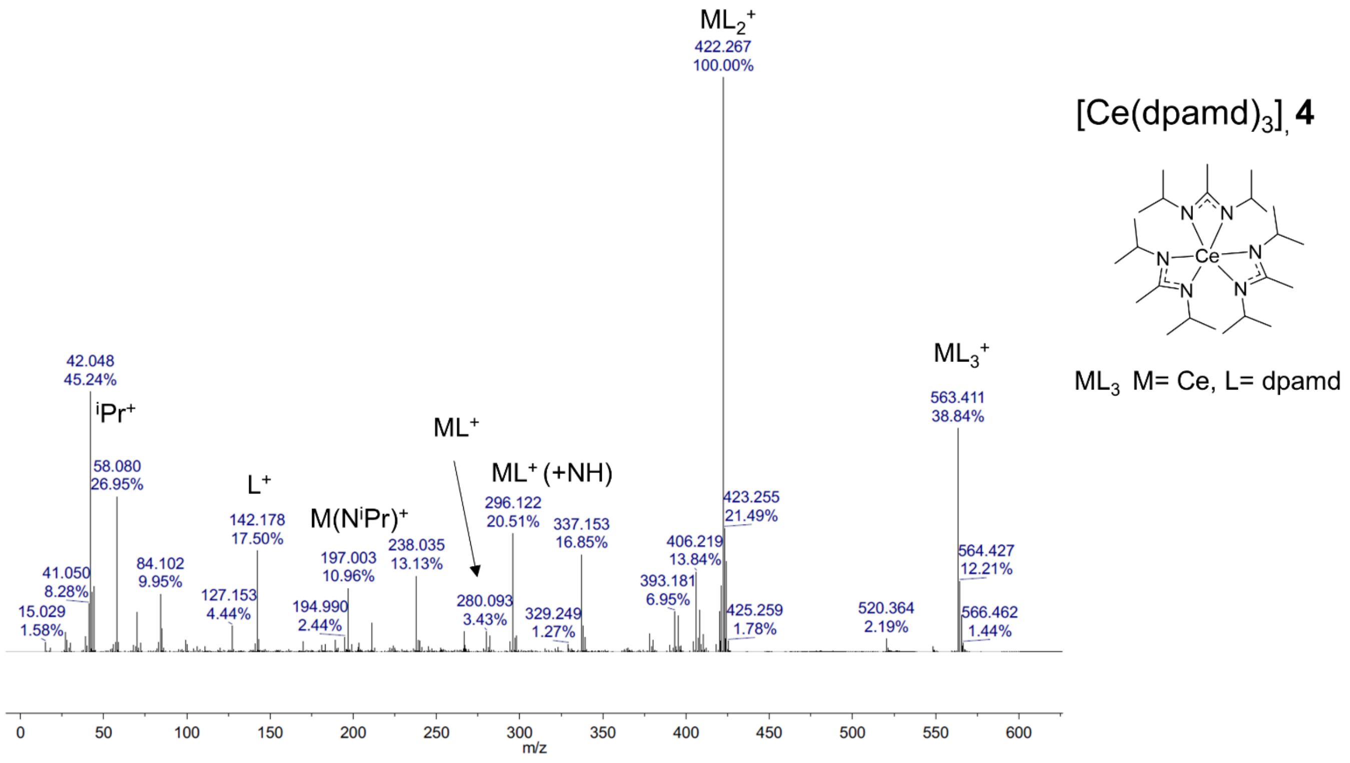

Figure S9 Mass spectrum of [Ce(dpamd)3] **4** (EI-MS, 70 eV).

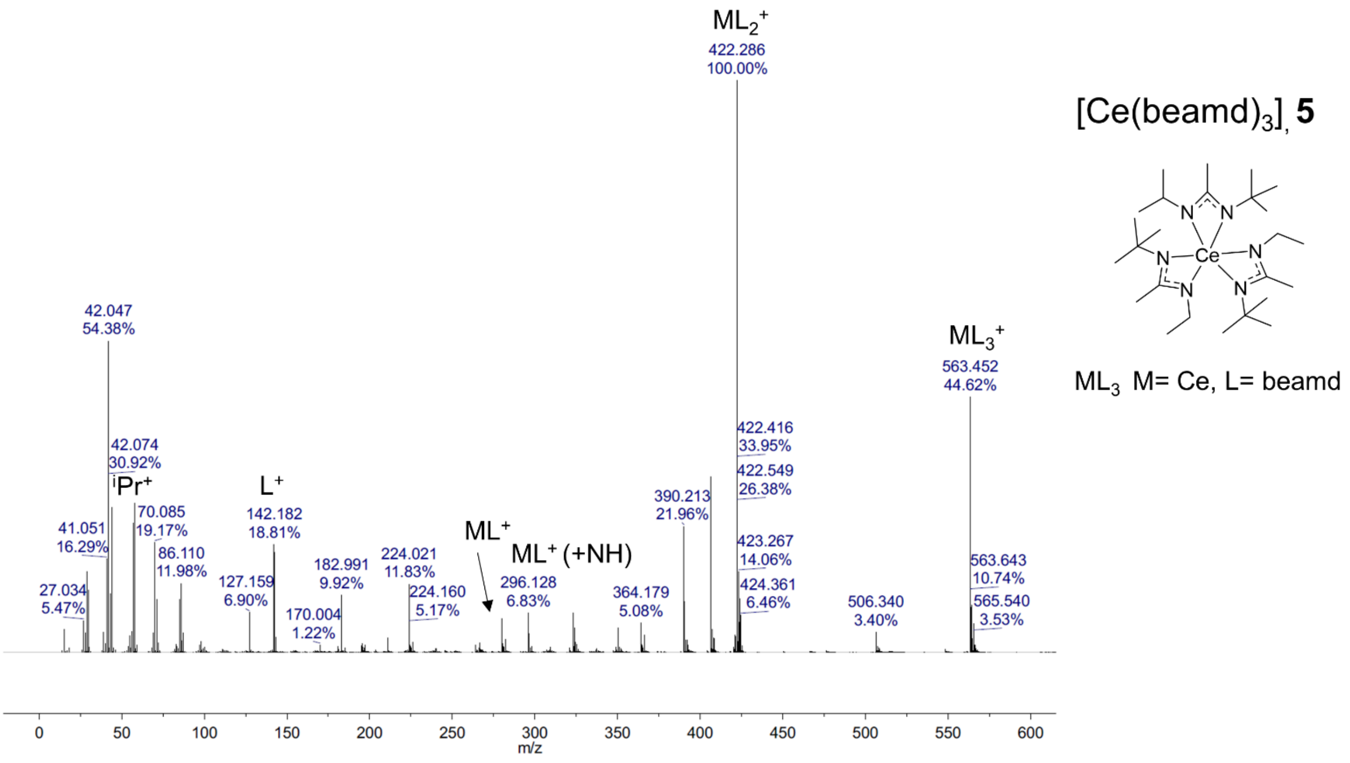

Figure S10 Mass spectrum of [Ce(beamd)3] **5** (EI-MS, 70 eV).

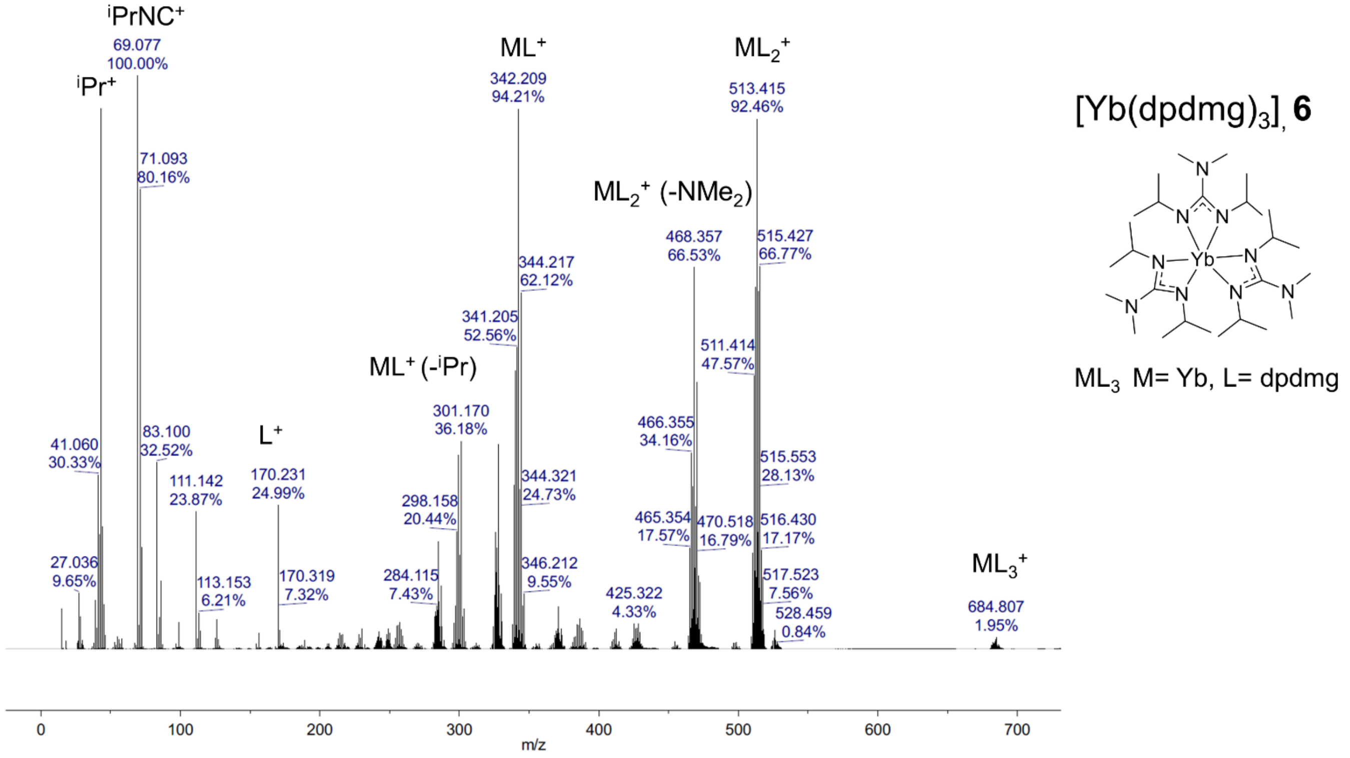

Figure S11 Mass spectrum of [Yb(dpdmg)3] **6** (EI-MS, 70 eV).

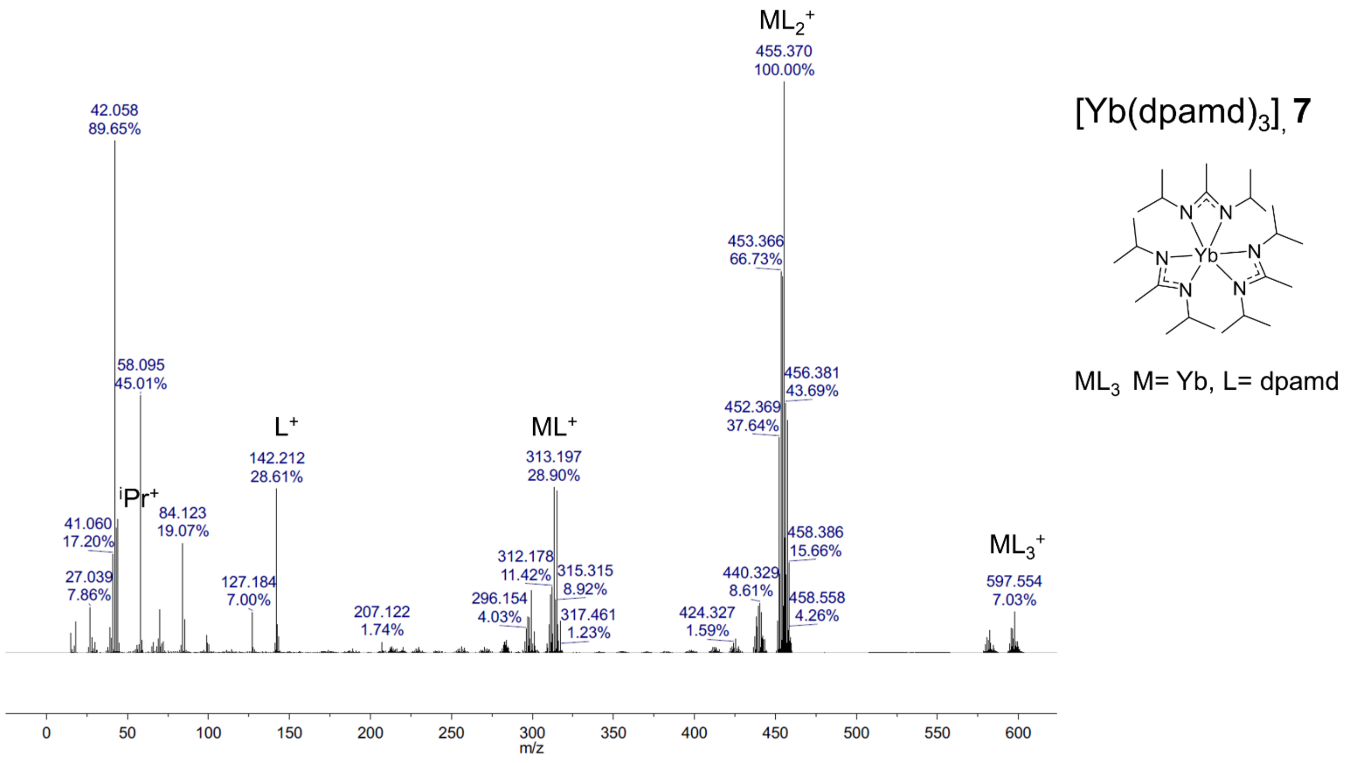

Figure S12 Mass spectrum of [Yb(dpamd)3] **7** (EI-MS, 70 eV).

Table S1 Crystallographic data for the complexes [Ce(dpdmg)$_3$], **1** and [Yb(dpdmg)$_3$], **6**.

	[Ce(dpdmg)$_3$], **1**	[Yb(dpdmg)$_3$], **6**
Empirical formula	$C_{27}H_{60}N_9Ce$	$C_{27}H_{60}N_9Yb$
Formula weight	650.96	683.88
Radiation	CuKα (λ = 1.54184)	MoKα (λ = 0.71073)
Temperature/K	100.01(10)	170(2)
Crystal system	monoclinic	Triclinic
Space group	$C\,2/c$	$P\bar{1}$
a/Å	18.8502(4)	10.7302(6)
b/Å	12.7341(3)	11.2059(6)
c/Å	28.9412(5)	16.2251(6)
α/°	90	91.495(4)
β/°	92.414(2)	95.702(4)
γ/°	90	115.411(6)
Volume/Å^3	6940.9(3)	1748.33(17)
Z	8	2
ρ_{calc} / g/cm^3	1.246	1.299
μ/mm^{-1}	10.341	2.703
F(000)	2744.0	710
Crystal size/mm^3	0.225 × 0.182 × 0.17	0.600 × 0.400 × 0.200
2Θ range for data collection/°	8.382 to 146.252	6.19 to 54.998
Index ranges	-20 ≤ h ≤ 22, -14 ≤ k ≤ 15, -34 ≤ l ≤ 35	-13 ≤ h ≤ 13, -14 ≤ k ≤ 14, -21 ≤ l ≤ 20
Reflections collected	13669	32548
Independent reflections	6740 [R_{int} = 0.0229, R_{sigma} = 0.0292]	7946 [R_{int} = 0.0557, R_{sigma} = 0.0500]
Data/restraints/parameters	6740/0/352	7946/0/352
Goodness-of-fit on F^2	1.064	1.054
Final R indexes [I>=2σ (I)]	R_1 = 0.0243, wR_2 = 0.0621	R_1 = 0.0293, wR_2 = 0.0628
Final R indexes [all data]	R_1 = 0.0263, wR_2 = 0.0633	R_1 = 0.0379, wR_2 = 0.0688
Largest diff. peak/hole / e Å^{-3}	0.51/-0.76	1.15/-0.76

Table S2 Summary of the thermal properties of the complexes **1** – **7**.

Complex	Onset of volatilization [°C]	Melting point [°C]	Residual weight [%]
**1** [Ce(dpdmg)$_3$]	90.1	104	25.2
**2** [Ce(bedmg)$_3$]	76.4	88	34.1
**3** [Ce(dpdeg)$_3$]	67.1	134	33.4
**4** [Ce(dpamd)$_3$]	70.8	-	15.5
**5** [Ce(beamd)$_3$]	76.4	50	15.5
**6** [Yb(dpdmg)$_3$]	155.3	110	20.8
**7** [Yb(dpamd)$_3$]	110.6	-	5.23

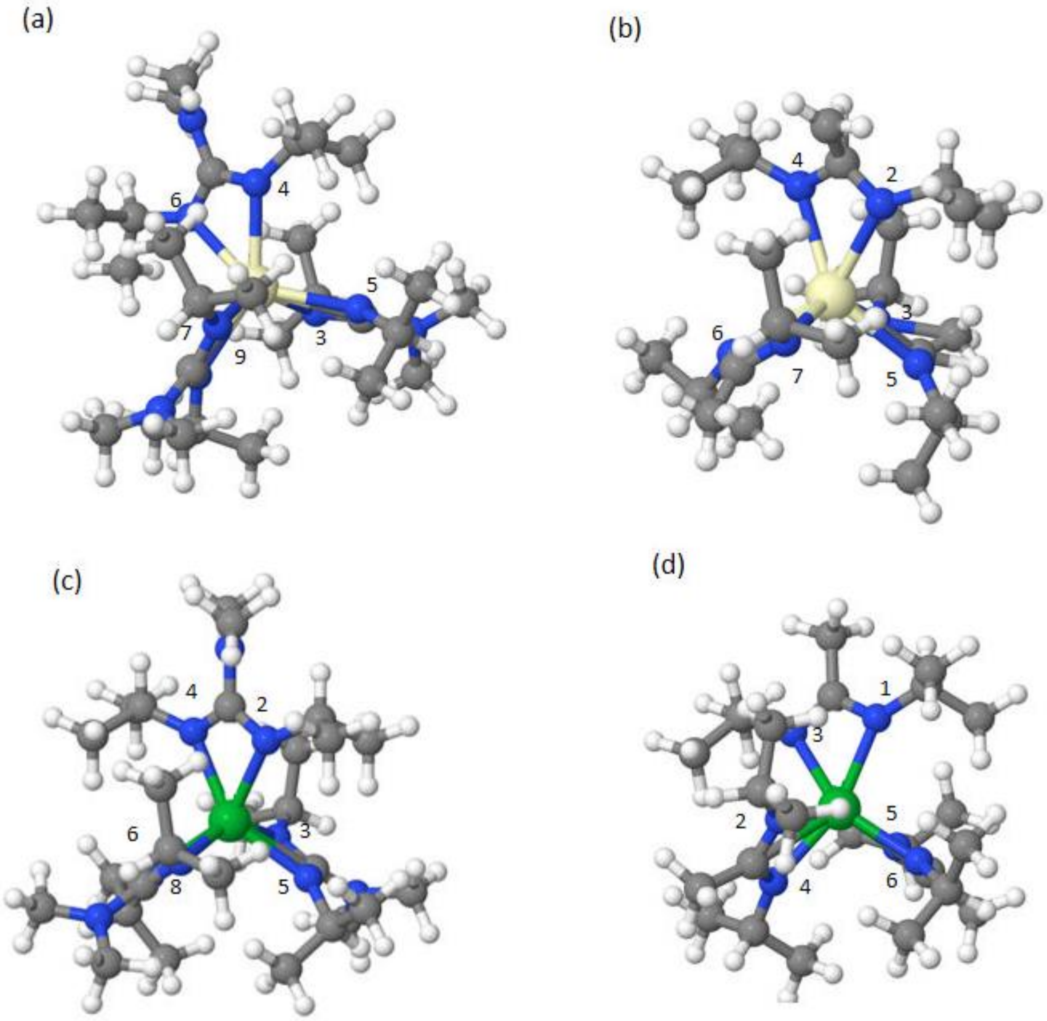

Figure S13 Atomic structures of (a) [Ce(dpdmg)$_3$] **1**, (b) [Ce(dpamd)$_3$] **4**, (c) [Yb(dpdmg)$_3$] **6**, and (d) [Yb(dpamd)$_3$] **7**. Cream: Cerium, Green: Ytterbium, Blue: Nitrogen, Gray: Carbon, White: Hydrogen. All the numbers labelled on Nitrogen atoms are according to Table S3.

Table S3 M-N distances of precursors in vacuum conditions

[Ce(dpdmg)3] **1**		[Ce(dpamd)3] **4**		[Yb(dpdmg)3] **6**		[Yb(dpamd)3] **7**	
Ce-N3	2.51 Å	Ce-N2	2.48 Å	Yb-N2	2.36 Å	Yb-N1	2.34 Å
Ce-N4	2.52 Å	Ce-N3	2.53 Å	Yb-N3	2.36 Å	Yb-N2	2.33 Å
Ce-N5	2.51 Å	Ce-N4	2.51 Å	Yb-N4	2.35 Å	Yb-N3	2.34 Å
Ce-N6	2.50 Å	Ce-N5	2.48 Å	Yb-N5	2.34 Å	Yb-N4	2.32 Å
Ce-N7	2.51 Å	Ce-N6	2.49 Å	Yb-N6	2.35 Å	Yb-N5	2.35 Å
Ce-N9	2.51Å	Ce-N7	2.51 Å	Yb-N8	2.35 Å	Yb-N6	2.34 Å

Table S4 N-M-N bite angle of the precursors from DFT.

[Ce(dpdmg)3] **1**		[Ce(dpamd)3] **4**		[Yb(dpdmg)3] **6**		[Yb(dpamd)3] **7**	
N4-Ce-N6	53.6°	N4-Ce-N2	53.9°	N3-Yb-N5	57.5°	N1-Yb-N3	57.3°
N5-Ce-N3	53.7°	N7-Ce-N6	53.9°	N4-Yb-N2	57.5°	N6-Yb-N5	57.5°
N9-Ce-N7	53.8°	N3-Ce-N5	53.5°	N8-Yb-N6	57.5°	N2-Yb-N4	57.7°

Table S5 M-O, O-N, O-O and M-N distances of precursors after the incorporation of oxygen.

[Ce(dpdmg)3] **1**		[Ce(dpamd)3] **4**		[Yb(dpdmg)3] **6**		[Yb(dpamd)3] **7**	
Ce-O1	2.32 Å	Ce-O1	2.29 Å	Yb-O1	2.83 Å	Yb-O1	2.38 Å
O1-N7	1.35 Å	O1-N3	1.34 Å	O1-O2	1.45 Å	O1-O2	1.46 Å
Ce-O2	1.82 Å	Ce-O2	1.82 Å	Yb-O1	2.15 Å	Yb-O2	2.16 Å
Ce-N3	2.71 Å	Ce-N2	2.73 Å	Yb-N2	2.54 Å	Yb-N1	2.41 Å
Ce-N4	2.53 Å	Ce-N4	2.78 Å	Yb-N3	2.30 Å	Yb-N2	2.33 Å
Ce-N5	2.77 Å	Ce-N5	2.64 Å	Yb-N5	2.40 Å	Yb-N3	2.30 Å
Ce-N6	2.61 Å	Ce-N6	2.48 Å	Yb-N6	2.42 Å	Yb-N4	2.39 Å
Ce-N9	2.61 Å	Ce-N7	2.59 Å	Yb-N8	2.32 Å	Yb-N5	2.53 Å

Table S6 M-OH and M-N distances of precursors after the incorporation of oxygen.

[Ce(dpdmg)3] **1**		[Ce(dpamd)3] **4**		[Yb(dpdmg)3] **6**		[Yb(dpamd)3] **7**	
Ce-OH	2.23 Å	Ce-OH	2.22 Å	Yb-OH	2.11 Å	Yb-OH	2.10 Å
Ce-N3	2.70 Å	Ce-N2	2.55 Å	Yb-N2	2.42 Å	Yb-N1	2.52 Å
Ce-N4	2.48 Å	Ce-N4	2.51 Å	Yb-N4	2.32 Å	Yb-N2	2.34 Å
Ce-N6	2.61 Å	Ce-N5	2.68 Å	Yb-N5	2.58 Å	Yb-N4	2.38 Å
Ce-N7	2.56 Å	Ce-N6	2.61 Å	Yb-N6	2.39 Å	Yb-N5	2.40 Å
Ce-N9	2.51 Å	Ce-N7	2.45 Å	Yb-N8	2.34 Å	Yb-N6	2.33 Å

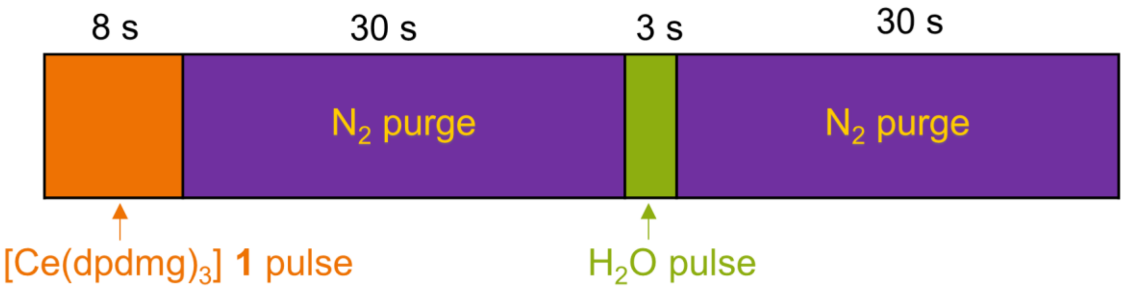

Figure S14: Schematic of pulse/purge sequence applied for thickness dependent studies at a deposition temperature of 160 °C.

Table S7 XPS peak assignment, positions and relative area of Ce 3d peaks found for a 42 nm thick CeO_2 film grown on Si(100). The areas of the different spin-orbital components are used to estimate the percentage of Ce^{3+} and Ce^{4+}.

Peak assignment	Ce species	Binding energy (eV)	Relative area (%)
**v°**	Ce^{3+}	882.0	9.3
**v**	Ce^{4+}	882.2	22.9
**v'**	Ce^{3+}	885.3	8.1
**v''**	Ce^{4+}	888.1	10.9
**v'''**	Ce^{4+}	898.1	17.1
**u°**	Ce^{3+}	900.1	3.2
**u**	Ce^{4+}	900.9	11.3
**u'**	Ce^{3+}	903.6	4.2
**u''**	Ce^{4+}	906.8	4.8
**u'''**	Ce^{4+}	916.6	9.2

Percentage of Ce_2O_3 = 24.8 %
Percentage of CeO_2 = 75.2 %
